# Therapeutic Potential of TLR8 Agonist GS‐9688 (Selgantolimod) in Chronic Hepatitis B: Remodeling of Antiviral and Regulatory Mediators

**DOI:** 10.1002/hep.31695

**Published:** 2021-06-20

**Authors:** Oliver E. Amin, Emily J. Colbeck, Stephane Daffis, Shahzada Khan, Dhivya Ramakrishnan, Divya Pattabiraman, Ruth Chu, Holly Micolochick Steuer, Sophie Lehar, Leanne Peiser, Adam Palazzo, Christian Frey, Jessica Davies, Hassan Javanbakht, William M.C. Rosenberg, Simon P. Fletcher, Mala K. Maini, Laura J. Pallett

**Affiliations:** ^1^ Division of Infection & Immunity Institute of Immunity & Transplantation University College London London United Kingdom; ^2^ Gilead Sciences Inc. Foster City CA; ^3^ Institute for Liver and Digestive Health University College London London United Kingdom; ^4^ Present address: Arbutus Biopharma Corporation Warminster PA; ^5^ Present address: Genentech Inc. South San Francisco CA; ^6^ Present address: Bristol Myers Squibb Seattle WA; ^7^ Present address: Ideaya Biosciences Inc. South San Francisco CA; ^8^ Present address: SQZ Biotechnologies Watertown MA

## Abstract

**Background and Aims:**

GS‐9688 (selgantolimod) is a toll‐like receptor 8 agonist in clinical development for the treatment of chronic hepatitis B (CHB). Antiviral activity of GS‐9688 has previously been evaluated *in vitro* in HBV‐infected hepatocytes and *in vivo* in the woodchuck model of CHB. Here we evaluated the potential of GS‐9688 to boost responses contributing to viral control and to modulate regulatory mediators.

**Approach and Results:**

We characterized the effect of GS‐9688 on immune cell subsets *in vitro* in peripheral blood mononuclear cells of healthy controls and patients with CHB. GS‐9688 activated dendritic cells and mononuclear phagocytes to produce IL‐12 and other immunomodulatory mediators, inducing a comparable cytokine profile in healthy controls and patients with CHB. GS‐9688 increased the frequency of activated natural killer (NK) cells, mucosal‐associated invariant T cells, CD4^+^ follicular helper T cells, and, in about 50% of patients, HBV‐specific CD8^+^ T cells expressing interferon‐γ. Moreover, *in vitro* stimulation with GS‐9688 induced NK‐cell expression of interferon‐γ and TNF‐α, and promoted hepatocyte lysis. We also assessed whether GS‐9688 inhibited immunosuppressive cell subsets that might enhance antiviral efficacy. Stimulation with GS‐9688 reduced the frequency of CD4^+^ regulatory T cells and monocytic myeloid‐derived suppressor cells (MDSCs). Residual MDSCs expressed higher levels of negative immune regulators, galectin‐9 and programmed death‐ligand 1. Conversely, GS‐9688 induced an expansion of immunoregulatory TNF‐related apoptosis‐inducing ligand^+^ NK cells and degranulation of arginase‐I^+^ polymorphonuclear MDSCs.

**Conclusions:**

GS‐9688 induces cytokines in human peripheral blood mononuclear cells that are able to activate antiviral effector function by multiple immune mediators (HBV‐specific CD8^+^ T cells, CD4^+^ follicular helper T cells, NK cells, and mucosal‐associated invariant T cells). Although reducing the frequency of some immunoregulatory subsets, it enhances the immunosuppressive potential of others, highlighting potential biomarkers and immunotherapeutic targets to optimize the antiviral efficacy of GS‐9688.

**Abbreviations**
cDC: conventional dendritic cell
CHB: chronic hepatitis B
FOXP3: forkhead box P3
HLA: human leukocyte antigen
ICOS: inducible T‐cell co‐stimulator
IFN: interferon
mAb: monoclonal antibody
MAIT: mucosal‐associated invariant T cell
MDSC: myeloid‐derived suppressor cells
MEC: minimal effective concentration
MFI: mean fluorescence intensity
M‐MDSC: monocytic‐MDSC
MNP: mononuclear phagocyte
NK: natural killer
PBMC: peripheral blood mononuclear cell
PD‐1: programmed cell death protein 1
pDC: plasmacytoid dendritic cell
PD‐L1: programmed death‐ligand 1
PMN‐MDSC: polymorphonuclear‐MDSC
REC: Research Ethics Committee
TFH: CD4+ follicular helper T cell
TLR: toll‐like receptor
TRAIL: TNF‐related apoptosis‐inducing ligand
T_REG_
: CD4+ regulatory T cells

Chronic hepatitis B (CHB) remains a global health concern with an estimated 260 million people infected worldwide. CHB causes more than 800,000 deaths a year due to HBV‐related complications such as cirrhosis and HCC.^(^ ^1^ ^)^ In the absence of novel treatment strategies, it is projected that new cases of CHB will rise to three million per year by 2030.^(^ ^2^ ^)^ Current approved therapies include long‐term antiviral suppression with nucleos(t)ide analogues and pegylated interferon‐α (IFN‐α). These treatments reduce viremia and improve patient outcomes but are rarely curative.^(^ ^3^ ^)^ As a consequence, there is a pressing need for novel immunotherapeutic strategies to supplement existing direct‐acting antivirals to achieve durable immune control.

Control of HBV is dependent on the coordinated action of both innate and adaptive immunity.^(^ ^4^ ^)^ A major obstacle to HBV clearance in CHB is a dysfunctional adaptive response, characterized by a profound state of immune exhaustion and HBV‐specific T‐cell depletion.^(^ ^5^, ^6^ ^)^ The mechanism by which this dysfunction occurs is multifaceted, but likely driven by a combination of ongoing high‐dose antigenic stimulation and the tolerogenic liver environment. Moreover, while the natural killer (NK) cell compartment can exert direct and indirect antiviral activity, they may also restrict effective antiviral immunity by deleting apoptosis‐prone HBV‐specific T cells. Specifically, NK cells in the HBV‐infected liver up‐regulate the death‐ligand TNF‐related apoptosis‐inducing ligand (TRAIL), enabling them to engage with and eliminate T cells expressing the reciprocal receptor, TRAIL‐receptor 2 (TRAIL‐R2).^(^ ^7^, ^8^ ^)^ Another mechanism contributing to the suppression of HBV immunity is the expansion of arginase^+^ polymorphonuclear myeloid‐derived suppressor cells (PMN‐MDSCs), which deplete key nutrients required by T cells for proliferation and function.^(^ ^9^ ^)^

One therapeutic approach in clinical development for CHB is to engage innate immune receptors such as toll‐like receptors (TLRs), which can exert both direct and indirect effects on the antiviral T‐cell and B‐cell response. A previous therapeutic strategy attempted to harness TLR7 signaling using the agonist GS‐9620 (vesatolimod). GS‐9620 induced sustained antiviral responses in animal models,^(^ ^10^, ^11^ ^)^ but failed to show therapeutic efficacy in patients with CHB.^(^ ^12^, ^13^ ^)^

More recently, attention has turned to TLR8 activation due to its anticipated ability to stimulate host immunity through the induction of pro‐inflammatory and immunomodulatory cytokines. Human TLR8 is predominantly expressed on the endosomal membrane of monocytes, macrophages, conventional dendritic cells (cDCs), and CD4^+^ regulatory T cells (T_REG_), and allows cells to respond to infection through detection of viral single‐stranded RNA.^(^ ^14^, ^15^, ^16^ ^)^ GS‐9688 (selgantolimod) is an oral selective small‐molecule agonist of TLR8 in development for CHB. *In vitro* studies have demonstrated that cytokines induced in human peripheral blood mononuclear cells (PBMCs) by GS‐9688 reduce viral parameters in HBV‐infected primary human hepatocytes.^(^ ^17^ ^)^ Furthermore, GS‐9688 treatment was well tolerated and induced a sustained antiviral response in a subset of woodchuck hepatitis virus–infected woodchucks.^(^ ^18^ ^)^ Although these preclinical studies provide some insight into the antiviral efficacy of GS‐9688, they did not provide insight into the mode of action of GS‐9688 on human immune cells.

Accordingly, in this study we have evaluated the immunomodulatory effects of GS‐9688 on various immune cells *in vitro* using freshly isolated peripheral leukocytes from healthy controls and patients with CHB, to understand its therapeutic potential for CHB.

## Experimental Procedures

### Ethical Approval

This study was approved by the local ethics board of Brighton & Sussex (Research Ethics Committee [REC] ref: 11/LO/0421), London Brent (REC ref: 17/LO/0266), and University College London–Royal Free Biobank (REC ref: 16/WA/0289). All participants gave written, informed consent. In select experiments, whole blood was obtained from AllCells (Alameda, CA) and the MT Group (Van Nuys, CA). In this instance, consent was obtained from the donor or donor’s legal next of kin using internal review board–approved authorizations. Study protocols conformed to the 1975 Declaration of Helsinki guidelines, and samples/data were stored in compliance with the Data Protection Act 1998 & Human Tissue Act 2004.

### Study Cohort

All participants were anti‐HCV and anti‐HIV antibody negative. Healthy controls were additionally anti‐HBV negative. Participants with CHB were stratified by HBV viral load (IU/mL; determined by real‐time PCR), HBsAg titer (IU/mL; determined by Architect; Abbott Laboratories, London, United Kingdom), HBeAg positivity, and serum alanine transaminase (ALT; IU/L) where appropriate (Supporting Table S1).

### Investigational Drug

GS‐9688 (selgantolimod) is a small‐molecule TLR8 agonist manufactured by Gilead Sciences Inc. (Foster City, CA).^(^ ^17^ ^)^ Concentrations used are denoted in the figures/figure legends.

### PBMC Isolation

PBMCs were isolated using Pancoll (PanBiotech GmbH, Aidenbach, Germany) or Ficoll (GE Healthcare Sciences, Chicago, IL) by density centrifugation from heparinized blood. Cells were washed and resuspended in Roswell Park Memorial Institute 1640 medium (Life Technologies, Carlsbad, CA) containing 10% vol/vol heat‐inactivated fetal bovine serum (HI‐FBS, Sigma‐Aldrich, St. Louis, MO; or Hyclone, GE Healthcare Sciences), 100 U/mL penicillin/streptomycin, 4‐(2‐hydroxyethyl)‐1‐piperazine ethanesulfonic acid, β‐mercaptoethanol, and essential and non‐essential amino acids (cRPMI; all Thermo Fisher Scientific, Waltham, MA).

### Measurement of Cytokines by Luminex

A total of 1 × 10^6^ PBMCs/well were seeded in 96‐well round‐bottom plates in 200 µL cRPMI with GS‐9688 or ≤0.1% DMSO vehicle control, at 37°C. After 24 hours, culture supernatants were harvested and stored at −80°C. Cytokine concentrations were determined by Luminex array (Bio‐Plex 200 System; Bio‐Rad Laboratories, Hercules, CA) or MAGPIX (Luminex Corporation, Austin, TX) with the following kits: Th1/Th2 cytokine panel (eBioscience, Inc., San Diego, CA), cytokine discovery fixed 45‐plex (Bio‐Techne, Minneapolis, MN) and a single‐plex IL‐12/IL‐23p40 (ProcartaPlex; eBioscience, Inc.).

### *In Vitro* Expansion and Detection of HBV‐Specific T Cells

Approximately 0.5‐1 ×10^6^ PBMCs/well were seeded in 96‐well round‐bottom plates in cRPMI for 7‐14 days, at 37°C. Cells were stimulated with 1 µg/mL pan‐genotypic overlapping 15‐mer peptides spanning the HBcAg overlapping peptide (OLP) (provided by Gilead Sciences Inc.) with GS‐9688 or ≤0.1% DMSO vehicle control from 0 days. Cells were maintained in 20 IU/mL recombinant human (rh)‐IL‐2 (Peprotech, London, United Kingdom) and continually supplemented throughout the expansion period. Cells were restimulated with HBV‐OLP for the final 16 hours in the presence of 1 µg/mL Brefeldin‐A (Sigma‐Aldrich) and Monensin (GolgiStop; BD Biosciences, San Jose, CA) at 37°C, before analysis by flow cytometry (described subsequently). Where appropriate, replicate wells were pooled before restimulation. Alternatively, HBV‐specific CD8^+^ T cells were expanded using 0.1 µg/mL ProMix HBV‐OLP, 15‐mer peptide pool of nine human leukocyte antigen (HLA)‐A2/A11/A24‐restricted peptides (ProImmune, Oxford, United Kingdom), or 1 µM HBV‐derived HLA‐A2‐restricted peptides (core FLPSDFFPSV; envelope FLLTRILTI, WLSLLVPFV, LLVPFVQWFV, and GLSPTVWLSV; polymerase GLSRYVARL and KLHLYSHPI [ProImmune]). HBV‐specific CD8^+^ T cells were detected using HLA‐specific multimers, pooled as appropriate for each patient HLA and multimer type. Briefly, after expansion, cells were resuspended in multimer wash buffer (1 × PBS with 2% human AB serum; Life Technologies), containing a pool of HLA‐restricted multimers (Supporting Table S2). Cells were stained with multimers for 15 minutes, at 37°C, before monoclonal antibody (mAb) staining for flow cytometric analysis.

### Multiparametric Flow Cytometry

For all flow cytometric analysis, cells were washed before staining in 1 × PBS. Cells were first stained with a fixable cell viability dye then stained with mAbs (Supporting Table S3) in brilliant violet buffer (BD Biosciences) for 30 minutes. Once stained, cells were washed and fixed with BDCytofix (BD Biosciences) for 20 minutes at 4°C. For intracellular antigens, cells were fixed and permeabilized using BDCytofix/Cytoperm and further stained with mAbs against intracellular antigens in 0.1% wt/vol Saponin (Sigma‐Aldrich). For intranuclear antigens, cells were fixed and permeabilized using the human forkhead box P3 (FOXP3) buffer (BD Biosciences), and further stained with mAbs targeting intranuclear antigens in 1 × PBS for 30 minutes at 4°C. Samples were acquired on a LSRFortessaX20, and data analyzed using FlowJo (v.10.4.1; BD Biosciences). Single stain controls and anti‐mouse immunoglobulin G (IgG) CompBeads (BD Biosciences) were used where appropriate.

### Assessment of Mononuclear Phagocytes, cDCs, and Plasmacytoid Dendritic Cell Phenotype and Function

A total of 1 × 10^6^ PBMCs/well were seeded in 96‐well flat‐bottom plates in cRPMI with GS‐9688 or ≤0.1% DMSO vehicle control at 37°C. After 2 hours, cells were treated with 50% vol/vol BDGolgiPlug (BD Biosciences) diluted in 1 × PBS and incubated at 37°C for 6 hours. Cells were then harvested and stained for flow cytometric analysis.

### Assessment of MAIT and NK Cell Phenotype and Function

A total of 0.5‐1 × 10^6^ PBMCs/well were seeded in 96‐well round‐bottom plates in cRPMI with GS‐9688 or ≤0.1% DMSO vehicle control at 37°C. After 18 hours, cells were resuspended in fresh cRPMI supplemented with 5 μg/mL Brefeldin‐A and 5 μg/mL BDGolgiStop and incubated for a further 5 hours. Cells were then harvested, stained with a panel of mAbs (Supporting Table S3), and analyzed by flow cytometry. For cytokine blocking, cells were treated with 10 µg/mL neutralizing antibodies against IL‐12p70 (clone:24910; R&D Systems, Inc., Minneapolis, MN), IL‐18 (clone 125‐2H; MBL USA Corp., Ottawa, IL), or isotype control IgG_1_ (clone 11711; R&D Systems, Inc.).

### HepG2 Lysis Assay

NK cell cytolytic activity was assessed using a fluorescence‐based killing assay (DELFIA ETDA Cytotoxicity Assay; PerkinElmer, Waltham, MA). PBMCs were treated with GS‐9688 or ≤0.1% DMSO vehicle control at 37°C. After 16 hours, NK cells were isolated by negative selection (StemCell Technologies, Kent, WA). HepG2 cells (HCC cell line [HB‐8065]; ATCC, Manassas, VA) were loaded with the intracellular‐specific fluorescence‐enhancing ligand BATDA and incubated for 30 minutes. HepG2 were co‐cultured with pretreated, purified NK cells at a 1:10 target‐to‐effector ratio in V‐bottomed plates and incubated for a further 2 hours. Culture supernatants were then harvested and extracellular release of BATDA was assessed by fluorescence activity using Europium solution, measured by a time‐resolved fluorometer (VICTOR, PerkinElmer).

### Assessment of T_REG_ and CD4+ Follicular Helper T‐Cell Frequency, Phenotype, and Function

A total of 0.5‐1 × 10^6^ PBMCs/well were seeded in 96‐well round‐bottom plates in cRPMI and stimulated with GS‐9688 or ≤0.1% DMSO vehicle control at 37°C for 7 days. Cells were maintained in 500 IU/mL rhIL‐2, continually supplemented throughout. After 7 days, cells were harvested and analyzed by flow cytometry.

### Assessment of MDSC Frequency, Phenotype, and Function

A total of 1 × 10^6^ freshly isolated PBMCs were stained to determine *ex vivo* frequencies of MDSC subsets by flow cytometry. To determine the effect of GS‐9688 on MDSC frequencies in the short term, 1‐2 × 10^6^ PBMCs/well were seeded in 48‐well plates in cRPMI and cultured for 24 hours with GS‐9688 or ≤0.1% DMSO vehicle control at 37°C. Following incubation, cells were harvested for analysis by flow cytometry. Culture supernatants were stored at −80° for analysis of extracellular arginase‐I by ELISA (Hycult Biotech, Uden, the Netherlands).

### Statistical Analysis

The E_max_ defines the maximum cytokine level induced by GS‐9688. The minimal effective concentration (MEC) defines the concentration of GS‐9688 corresponding to three‐fold induction of cytokine above background (DMSO control). EC_50_ defines the concentration of GS‐9688 giving 50% of the maximal response. MEC and EC_50_ were calculated using the Pipeline Pilot v.9.2 (BIOVIA, San Diego, CA). All statistical analyses were performed in Prism (v.7.0e; GraphPad Software, San Diego, CA); tests used are indicated in figure legends (Wilcoxon signed‐rank *t* test; Kruskal‐Wallis test; Friedman test [ANOVA] with Dunn’s *post hoc* test; and ANOVA with Sidak’s *post hoc* test). All tests were carried out as two‐tailed tests. Significant differences are denoted in all figures and defined as *P < 0.05, **P < 0.01, ***P < 0.001, and ****P < 0.0001.

## Results

### *In Vitro* Stimulation of Human PBMC From Healthy Controls and Patients With CHB With GS‐9688 Produces Immunomodulatory Mediators

**FIG. 1:**
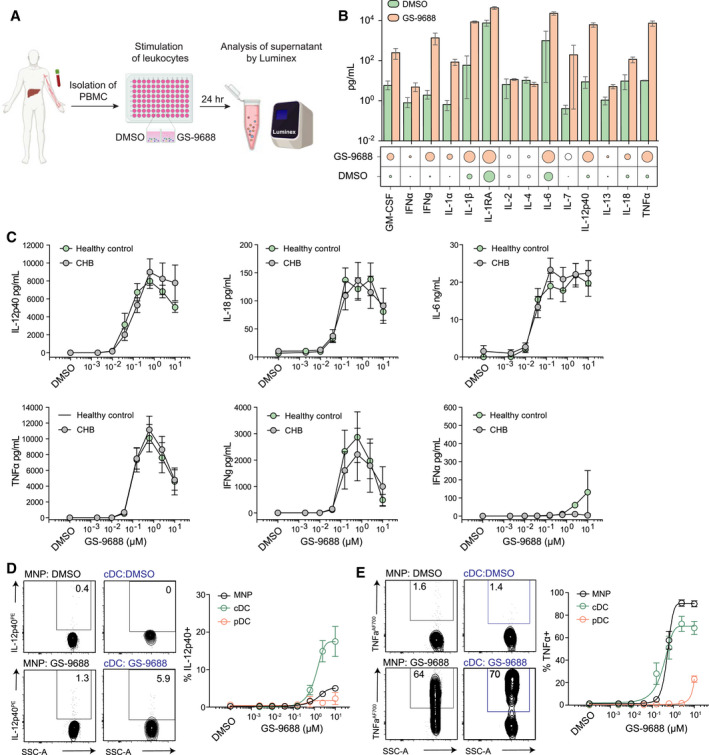
*In vitro* stimulation with GS‐9688 induces secretion of immunomodulatory cytokines by cDCs and MNPs. (A) Experimental design: evaluation of GS‐9688‐induced soluble mediators by multiplex array. (B) Concentration of cytokines from PBMC culture supernatants of healthy controls stimulated for 24 hours with 0.156 µM GS‐9688 or vehicle control (DMSO). Bars represents mean ± SEM; circle radius represents absolute expression; and filled‐in circles represent a significant difference between treatment groups (n = 10; Mann‐Whitney U test). (C) Levels of IL‐12p40/IL‐18/IL‐6/TNF‐α/IFN‐γ/IFN‐α following GS‐9688 treatment, serial‐diluted from 10 µM, from PBMCs isolated from healthy controls (n = 10) and patients with CHB (n = 10). Data indicate the mean ± SEM. Representative flow cytometric plots (left) and percentage of IL‐12p40^+^ cells (D) and TNFα+ cells (E) by circulating MNPs (Lin1^+^CD14^+^), cDCs (Lin1^−^HLA‐DR^+^CD123^−^CD11c^+^), and pDCs (Lin1^−^HLA‐DR^+^CD123^+^CD11c^−^) from GS‐9688 treated healthy control PBMC (n = 6). Data represent the mean ± SEM. Abbreviations: GM‐CSF, granulocyte‐macrophage colony‐stimulating factor; SSC‐A, side scatter–area.

To investigate the therapeutic potential of the TLR8 agonist GS‐9688, we first assessed its *in vitro* activity and potency using human PBMCs isolated from 10 healthy controls. PBMCs were stimulated with GS‐9688, and cell culture supernatants were evaluated by Luminex array (Fig. 1A). Among the cytokines tested, GS‐9688 induced the immunomodulatory cytokine IL‐12p40, in addition to the antiviral cytokines IL‐6, TNF‐α, and interferon‐g (IFN‐γ) (Fig. 1B and Supporting Fig. S1A). GS‐9688‐dependent cytokine induction occurred in a dose‐dependent manner, with a mean MEC and EC_50_ of 29/217 nM, 54/326 nM, and 55/267 nM for IL‐12p40, TNF‐α and IFN‐γ, respectively (Fig. 1C and Supporting Fig. S1A). Additionally, GS‐9688 induced the production of the pro‐inflammatory cytokines IL‐1α, IL‐1β, and the anti‐inflammatory mediator IL‐1RA (Fig. 1B,C and Supporting Fig. S1A). Conversely, GS‐9688 induced little to no IFN‐α, a prototypical cytokine predominantly produced following engagement of TLR7,^(^ ^19^, ^20^ ^)^ consistent with its selectivity for human TLR8^(^ ^17^ ^)^ (Fig. 1B,C and Supporting Fig. S1A). Importantly, there was no significant difference in response in PBMCs from 10 age‐matched patients with CHB, with comparable MEC, EC_50_, and E_max_ for all cytokines tested (Fig. 1C and Supporting Fig. S1A).

### GS‐9688 Activates cDCs and Mononuclear Phagocytes to Produce IL‐12p40 and TNF‐α

To investigate the cellular source of relevant immunomodulatory cytokines, we examined the effect of GS‐9688 on circulating myeloid cells. Human TLR8 is expressed primarily by cDCs and mononuclear phagocytes (MNPs), but is absent on plasmacytoid dendritic cells (pDCs).^(^ ^14^, ^15^, ^16^ ^)^ Short‐term stimulation of PBMCs with GS‐9688 increased production of IL‐12p40 by Lin1^−^HLA‐DR^+^CD123^−^CD11c^+^ cDCs, in a dose‐dependent manner (Fig. 1D). Likewise, TNF‐α was produced by cDCs and Lin1^+^CD14^+^ MNPs in response to GS‐9688 (Fig. 1E). The cDCs, pDCs, and MNPs produced minimal IFN‐α when treated with GS‐9688, consistent with selective activation of TLR8 by GS‐9688 (Supporting Fig. S1B). The activation and induction of immunomodulatory cytokines was supported by augmented expression of the activation marker CD40 on both cDCs and MNPs (Supporting Fig. S1C). Collectively, these data demonstrate that GS‐9688 activates cDCs and MNPs and is a potent inducer of the immunomodulatory cytokines IL‐12 and TNF‐α *in vitro*.

### Frequencies of Functional HBV‐Specific CD8^+^ T Cells Increase in a Subset of Patients With CHB After *In Vitro* Stimulation With GS‐9688

**FIG. 2:**
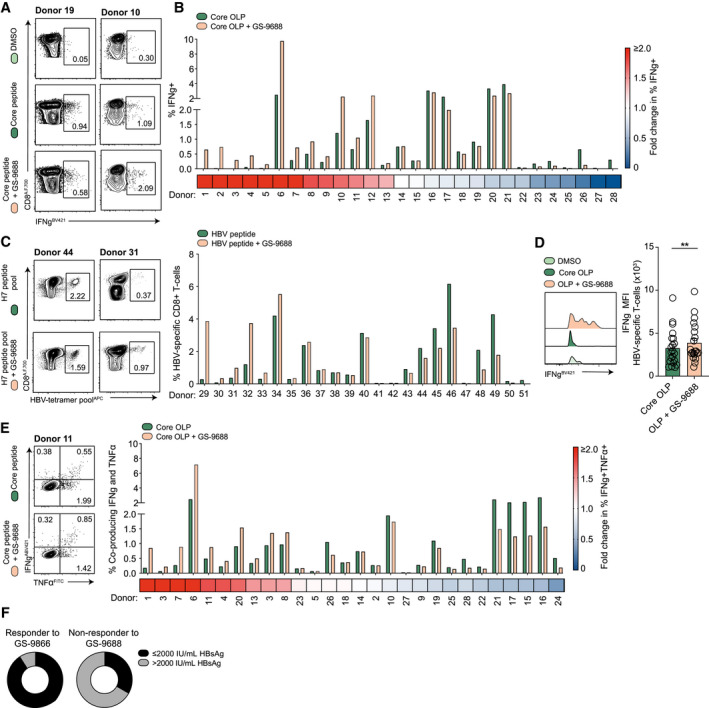
GS‐9688 increases HBV‐specific CD8^+^ T‐cell frequency and function in a subset of patients with CHB. HBV‐specific CD8^+^ T cells were expanded from PBMCs by stimulation with pan‐genotypic, overlapping peptides spanning the HBV‐core protein (OLP) in the presence of 0.1 µM GS‐9688 or vehicle control (DMSO). Representative flow cytometric plots of a “responder” and “non‐responder” to GS‐9688 treatment (A) and percentage of IFN‐γ‐producing HBV‐specific CD8^+^ T cells for individual patients (B). The percentage of IFN‐γ produced by DMSO‐treated CD8^+^ T cells was subtracted to determine HBV‐specific cytokine production (n = 28). Heat map denotes fold change in percentage of IFN‐γ production with GS‐9688. (C) Representative flow cytometric plots (left) and percentage of HBV‐specific CD8^+^ T cells identified by staining with a panel of immunodominant HBV‐specific HLA‐restricted multimers (see Supporting Table S2) after *in vitro* expansion with a pool of HBV‐derived HLA‐restricted peptides ± 0.156 µM GS‐9688 or DMSO (n = 23; Wilcoxon Signed‐rank *t* test). (D) MFI of IFN‐γ produced in response to HBV‐core OLP (n = 21). (E) Percentage of CD8^+^ T cells co‐producing IFN‐γ and TNF‐α in response to HBV‐core OLP (n = 28). (F) Stratification of patient non‐responders and responders (defined as a ≥1.2‐fold increase in percentage of IFN‐γ^+^ HBV‐specific CD8^+^ T cells in response to HBV‐core OLP in the presence of 0.1 µM GS‐9688) by baseline HBsAg titer (n = 23). Clinical characteristics of all patients with CHB are detailed in Supporting Table S1. Error bars represent the mean ± SEM. **P < 0.01.

CD8^+^ T cells are critical for the control of HBV infection, mediated in part through their capacity for noncytolytic inhibition of HBV replication by the secretion of antiviral cytokines.^(^ ^21^, ^22^, ^23^ ^)^ It is increasingly recognized that local cytokine environments can shape adaptive immune responses. Therefore, we tested the capacity of the cytokines induced by GS‐9688 to indirectly modulate the frequency of functional HBV‐specific CD8^+^ T cells in response to HBV‐OLP using PBMCs isolated from patients with CHB (clinical parameters at the time of sampling in Supporting Table S1 and gating strategy in Supporting Fig. S2A). In 13 of 28 patients examined, treatment with GS‐9688 increased the frequency of CD8^+^ T cells producing the antiviral cytokine IFN‐γ in response to HBcAg (Fig. 2A,B). A similar proportion of patients showed no increase in the number of IFN‐γ^+^ HBV‐specific CD8^+^ T cells following stimulation with GS‐9688 (Fig. 2B). Interestingly, *de novo* IFN‐γ responses induced by GS‐9688 were detected in 3 patients who lacked a detectable HBV‐specific response to HBV‐core OLP stimulation alone. Similarly, when using a panel of HLA‐restricted peptide multimers (Supporting Table S2) to detect HBV‐specific CD8^+^ T cells after peptide expansion, the response to GS‐9688 was heterogenous. Here, 9 of 23 patients showed a substantial increase in the proportion of detectable HBV‐specific CD8^+^ T cells (Fig. 2C). Notably, the mean fluorescence intensity (MFI) of IFN‐γ from the HBV‐specific IFN‐γ^+^ CD8^+^ T‐cell pool showed a modest increase, suggesting a greater production of this antiviral cytokine per cell in response to HBV‐core OLP in the presence of GS‐9688 (Fig. 2D). Furthermore, when assessing the frequency of polyfunctional CD8^+^ T cells, we observed an increase in the proportion of CD8^+^ T cells producing both IFN‐γ and TNF‐α in 10 patients (36%; Fig. 2E). In contrast, the percentage of CD8^+^ T cells degranulating in response to HBV‐core OLP only increased in 3 (16%) patients following treatment with GS‐9688 (Supporting Fig. S2B). Taken together, these data support the potential of GS‐9688 to enhance the noncytolytic effector function of pre‐existing endogenous HBV‐specific CD8^+^ T cells, and to increase the frequency of antiviral T cells in a subset of patients.

Finally, we explored whether the heterogeneity in the magnitude of the HBV‐specific T‐cell response to GS‐9688 was associated with any clinical parameters in our cohort. In doing so, we noted that a baseline HBsAg titer less than 2,000 IU/mL was associated with an increase in the proportion of IFN‐γ‐producing HBV‐specific T cells in the presence of GS‐9688 (Fig. 2F). In contrast, most patients denoted “non‐responders” (who did not have an increase in the frequency of IFN‐γ^+^ HBV‐specific CD8^+^ T cells with GS‐9688) had a baseline HBsAg titer greater than 2,000 IU/mL (Fig. 2F). No clear association in responsiveness was seen with serum ALT levels or HBV viral load (Supporting Fig. S2C).

### Treatment of PBMCs With GS‐9688 Potentiates the Activation and Function of Antiviral Effectors

Innate effector cells such as NK cells play a dual role in the setting of viral infection. Although these immune mediators can exert antiviral activity by direct or indirect effects (e.g., through modulation of T‐cell responses), emerging data underscore mechanisms by which they limit antiviral responses through the inhibition or killing of antigen‐specific T cells. In CHB, NK cells can also become defective in their capacity to produce IFN‐γ and TNF‐α, resulting in an inability to effectively exert noncytolytic activity in the HBV‐infected liver.^(^ ^24^, ^25^ ^)^

**FIG. 3:**
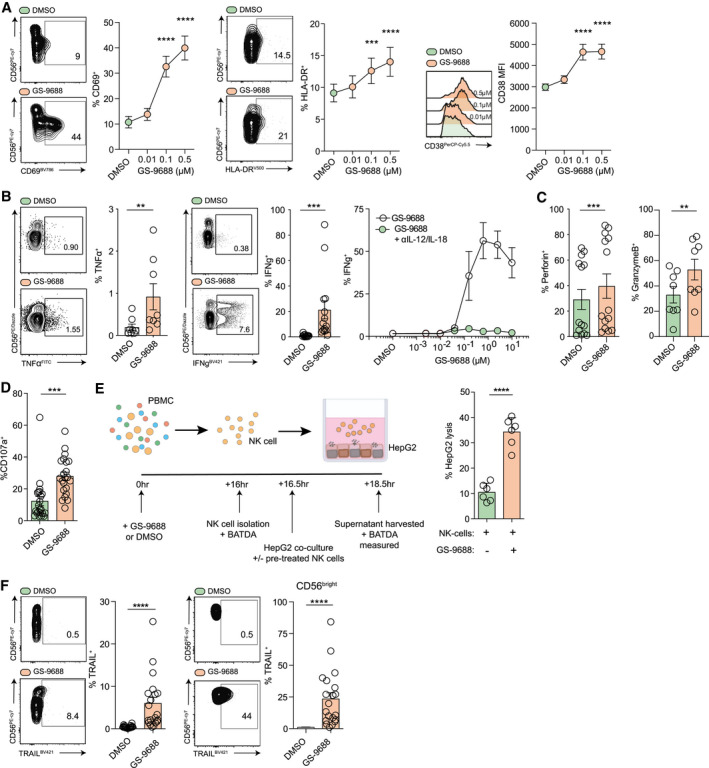
*In vitro* stimulation with GS‐9688 increases the cytolytic and noncytolytic potential of NK cells, while increasing TRAIL expression on the CD56^bright^ subset. PBMCs from healthy controls (HC) or patients with CHB were treated *in vitro* with GS‐9688 (dose range or single dose) or vehicle control (DMSO) for 24 hours. (A) Representative flow cytometric plots, percentages of CD69 and HLA‐DR, and MFI of CD38 expression (CHB; all n = 20; Friedman test [ANOVA] with a Dunn’s multiple comparisons test). (B) Percentage of TNF‐α^+^ (0.156 µM; HC; n = 8) and IFN‐γ^+^ (0.156 µM; HC; n = 14; Wilcoxon signed‐rank *t* test) ± neutralization of IL‐12/IL‐18 or isotype control (0.156 µM; HC; n = 6; Wilcoxon signed‐rank *t* test). (C) Percentage of perforin^+^ (0.156 µM; HC; n = 14) and granzyme B (0.156 µM; HC; n = 8; Wilcoxon signed‐rank *t* test). (D) Percentage of CD107a^+^ (0.156 µM; HC; n = 22; Wilcoxon signed‐rank *t* test) on global CD3^−^CD56^+^ NK cells. (E) Schematic of experimental setup and percentage of cell lysis of HepG2 hepatoma cell line (target cell) following co‐culture with purified NK cells pretreated with DMSO or GS‐9688 (0.2 µM; HC; n = 6; Wilcoxon signed‐rank *t* test). (F) Representative flow cytometric plots (left) and percentage of TRAIL expression on global CD3^−^CD56^+^ NK cells and CD56^bright^ NK cells (0.1 µM; CHB; n = 20; Wilcoxon signed‐rank *t* test). Error bars represent the mean ± SEM. **P < 0.01; ***P < 0.001; ****P < 0.0001.

To address the functional impact of GS‐9688 on NK cells *in vitro*, we determined the activation status of the global NK cell population in response to GS‐9688 treatment of PBMCs from healthy controls and patients with CHB. GS‐9688 activated NK cells in a dose‐dependent manner, increasing the expression of the activation markers CD69, HLA‐DR, and CD38 (gating strategy; Supporting Fig. S3A and Fig. 3A). Activation was evident on both the immature cytokine producing CD56^bright^ population, which has been described to be preferentially enriched in the liver,^(^ ^26^ ^)^ and the cytotoxic, more mature CD56^dim^ subset (Supporting Fig. S3B,C). In line with their activation, treatment with GS‐9688 augmented the noncytolytic effector function of NK cells, confirmed by an increased production of TNF‐α and IFN‐γ (Fig. 3B). As expected, the increase in cytokine production driven by GS‐9688 was largely attributable to the CD56^bright^ population (Supporting Fig. S3D). NK cells do not express TLR8 but can be activated by TLR8 agonists through the production of IL‐12 and IL‐18.^(^ ^27^ ^)^ Consistent with these data, neutralization of IL‐12/IL‐18 abrogated the IFN‐γ production seen in response to GS‐9688 treatment in the 6 individuals tested, at all concentrations of GS‐9688 (Fig. 3B). This finding supports a role for GS‐9688‐induced cytokines in promoting NK‐cell antiviral function.

Although IFN‐γ production by NK cells can promote antiviral T‐cell responses and inhibit viral replication in hepatocytes, additional anti‐HBV activity of NK cells is attributable to their ability to directly eliminate infected hepatocytes.^(^ ^25^ ^)^ In line with this, GS‐9688 enhanced the cytolytic capacity of NK cells, as indicated by increased expression of perforin and granzyme B (Fig. 3C) and the propensity for degranulation, as denoted by CD107a mobilization (Fig. 3D). Moreover, GS‐9688‐treated, purified NK cells also increased lysis of the HepG2 hepatoma cell line (Fig. 3E). Collectively, these data demonstrate that GS‐9688 enhances both the noncytolytic and cytolytic effector functions of NK cells *in vitro*.

Although a role for MAITs (non‐classical T cells characterized by expression of the invariant chain Vα7.2) in CHB remains unclear, they are highly enriched in the liver and are potent producers of IFN‐γ in response to TLR8 agonism.^(^ ^28^ ^)^ GS‐9688 activated MAITs in PBMCs, increasing the expression of IFN‐γ and granzyme B (Supporting Fig. S4A‐C). Analogous to NK cells, this increase in the noncytolytic and cytolytic potential of MAITs by GS‐9688 was IL‐12/IL‐18‐dependent in 2 healthy controls (Supporting Fig. S4B,C).

In CHB, NK cells can also mediate negative regulatory effects by up‐regulating death ligands such as TRAIL to kill HBV‐specific T cells selectively expressing TRAIL‐R2.^(^ ^7^, ^8^ ^)^ Furthermore, TRAIL‐expressing NK cells have the potential to directly kill hepatocytes.^(^ ^7^ ^)^ GS‐9688 treatment induced a significant increase in TRAIL‐expressing NK cells (Fig. 3F), which was most evident on the CD56^bright^ subset (Fig. 3F and Supporting Fig. S3E).

Together, these data demonstrate that GS‐9688 can enhance the cytolytic and noncytolytic activity of multiple innate effectors *in vitro*.

### *In Vitro* GS‐9688 Reduces the Frequency of T_REG_ and Increases the Frequency of Circulating Follicular Helper CD4^+^ T Cells

Follicular helper CD4^+^ T cells (T_FH_) play a key role in promoting adaptive immunity through the generation of long‐lived plasma cells.^(^ ^29^, ^30^ ^)^ In contrast, CD4^+^ T_REG_ cells are potent immunosuppressors able to down‐regulate adaptive immunity, with previous studies reporting increases in the proportion of circulating and liver‐resident T_REG_ in patients, that actively inhibit the antiviral response and contributes to chronicity in CHB.^(^ ^31^ ^)^ Importantly, recent studies have highlighted the capacity of TLR8 agonists to both reduce the suppressive capacity of T_REG_ in a model of antitumor immunity^(^ ^15^ ^)^ and enhance T_FH_ differentiation.^(^ ^32^ ^)^ Thus, GS‐9688 was examined for its effect on the frequency and phenotype of circulating T_FH_ (cT_FH_) and T_REG_ cells by quantifying their proportions in PBMCs isolated from patients with CHB or healthy controls (T_REG_: CD4^+^CD25^hi^CD127^lo^FOXP3^+^; cT_FH_: CD4^+^CXCR5^+^PD‐1^+^; gating strategy found in Supporting Fig. S5A) after *in vitro* culture.

**FIG. 4:**
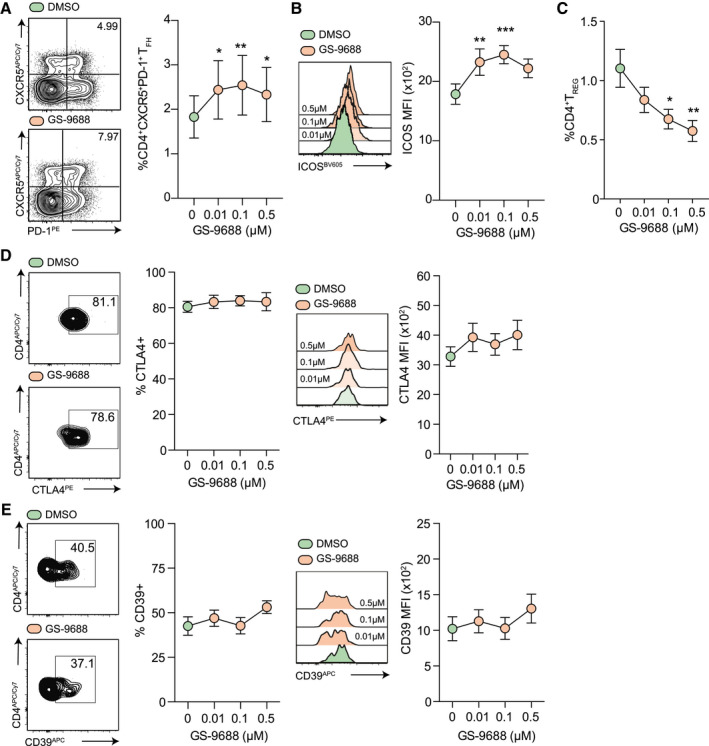
GS‐9688 increases the frequency of cT_FH_ and decreases the frequency of T_REG_. (A) Representative flow cytometric plots (left) and percentage of cT_FH_ (CD4^+^CXCR5^+^PD‐1^+^; healthy controls (HC); n = 12) as a proportion of global CD3^+^CD8^−^CD4^+^ T cells after 7 days of *in vitro* stimulation with GS‐9688 (dose range). Representative flow cytometric plots and summary data depicting ICOS expression (MFI; n = 12) (B) and percentage of CD4^+^ T_REG_ (CD4^+^CD127^lo^CD25^hi^FOXP3^+^; CHB/HC; n = 37) as a proportion of global CD3^+^CD8^−^CD4^+^ T cells after *in vitro* stimulation with GS‐9688 (dose range) (C). Representative flow cytometric plots and summary data depicting CTLA‐4 (percentage and MFI; n = 8/14) (D) and CD39 (percentage and MFI; n = 8/14) (E). Data represent the mean ± SEM. *P < 0.5. Freidman test (ANOVA) with a Dunn’s *post hoc* multiple comparisons test compared with DMSO control. Abbreviation: CTLA‐4, cytotoxic T‐lymphocyte‐associated protein 4.

Although the overall proportion of CD4^+^ T cells remained unchanged (Supporting Fig. S5B), there was an increase in the frequency of cT_FH_ in response to GS‐9688 at all doses tested (Fig. 4A). Furthermore, GS‐9688 altered the phenotype of cT_FH_ by increasing the expression of inducible T‐cell co‐stimulator (ICOS), a marker for T_FH_ differentiation and function^(^ ^33^ ^)^ (Fig. 4B). Conversely, treatment with GS‐9688 induced a dose‐dependent decrease in the frequency of circulating T_REG_ (Fig. 4C). Despite reducing the frequency of T_REG_, GS‐9688 had little impact on the immunosuppressive potential of the remaining T_REG_ population on the basis of unaltered expression of the negative regulator, cytotoxic T‐lymphocyte‐associated protein 4 (CTLA4; Fig. 4D) and CD39, the rate‐limiting enzyme in the generation of immunosuppressive adenosine (Fig. 4E). Although the potential for suppression by the remaining T_REG_ population was unaffected by GS‐9688, the ability of GS‐9688 to decrease the frequency of T_REG_ suggests that it may act to reduce the overall capacity of T_REG_ to limit HBV‐specific T‐cell responses *in vivo*.

### GS‐9688 Significantly Decreases Monocytic MDSC Frequencies, While Triggering a Release of Arginase‐I From PMN‐MDSCs

MDSCs are immature myeloid progenitors that exert potent immune regulation through the manipulation of nutrient availability and expression of inhibitory ligands like programmed death‐ligand 1 (PD‐L1).^(^ ^34^ ^)^ The MDSC compartment consists of two populations, polymorphonuclear MDSCs (PMN‐MDSCs) and monocytic MDSCs (M‐MDSCs).^(^ ^34^ ^)^ Arginase‐I expressing PMN‐MDSCs accumulate in patients with CHB, where they limit the proliferation and function of bystander and HBV‐specific T cells.^(^ ^9^ ^)^ Different TLR8 agonists were recently shown to reduce M‐MDSC frequencies *in vitro* by selectively inducing their apoptosis and limiting their immunosuppressive activity.^(^ ^35^, ^36^ ^)^ Consequently, we assessed the capacity of GS‐9688 to modulate the frequency and functionality of MDSC.

**FIG. 5:**
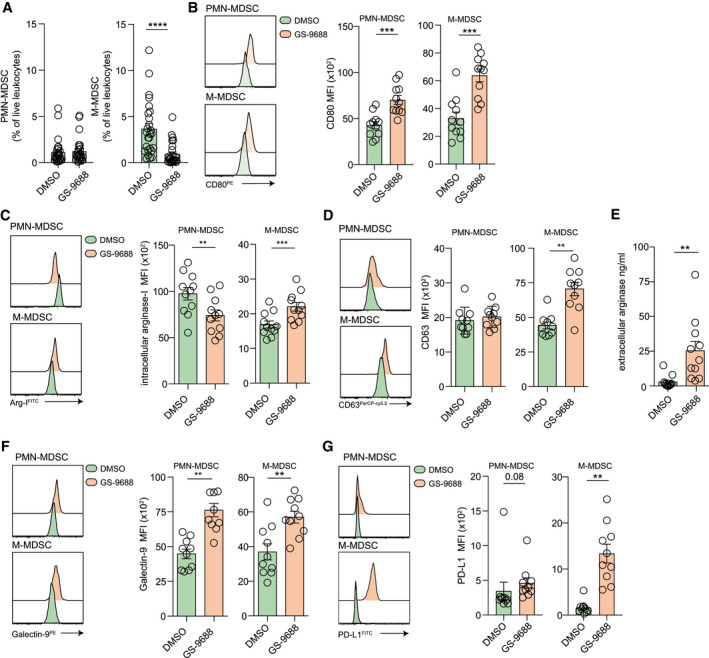
GS‐9688 skews the balance of MDSC subsets and alters their immunosuppressive potential in patients with CHB. PBMCs isolated from patients with CHB were stimulated with a single dose of 0.1 µM GS‐9688 or vehicle control (DMSO) for 18 hours. (A) Percentage of PMN‐MDSCs (CD11b^+^CD33^+^HLA‐DR^lo^CD14^−^CD15^+^) and M‐MDSCs (CD11b^+^CD33^+^HLA‐DR^lo^CD14^+^CD15^−^) as a percentage of total live leukocytes (n = 26; Wilcoxon signed‐rank *t* test). PMN‐MDSC and M‐MDSC expression of CD80 (MFI; n = 11) (B), intracellular arginase‐I (C), and CD63 (MFI; n = 10) (D) (all Wilcoxon signed‐rank *t* tests). (E) Extracellular arginase‐I concentration released into cell culture supernatant measured by ELISA (n = 11). PMN‐MDSC and M‐MDSC expression of galectin‐9 (MFI; n = 10) (F) and PD‐L1 (MFI; n = 10) (G) (both Wilcoxon signed‐rank *t* tests). Error bars represent the mean ± SEM. **P < 0.01; ***P < 0.001; ****P < 0.0001.

First, we assessed the effect of GS‐9688 on the frequency of MDSC subsets in patients with CHB, defining PMN‐MDSCs as CD11b^+^CD33^+^HLA‐DR^lo^CD14^−^CD15^+^ and M‐MDSCs as CD11b^+^CD33^+^HLA‐DR^lo^CD14^+^CD15^−^ (gating strategy found in Supporting Fig. S6A). The *ex vivo* frequency of each MDSC subset was quantified as a percentage of the immature myeloid compartment (CD11b^hi^CD33^+^), revealing that M‐MDSCs are the predominant subset, accounting for approximately 45% of immature myeloid precursors (Supporting Fig. S6B). In line with previous reports with other TLR8 agonists, short‐term culture with GS‐9688 led to a reduction in the frequency of M‐MDSCs (Fig. 5A and Supporting Fig. S6C). Although we noted a marked reduction in M‐MDSCs, this coincided with a relative increase in the proportion of immature myeloid cells with a PMN‐MDSC phenotype (Supporting Fig. S6C), the absolute frequency of PMN‐MDSCs remained unchanged when evaluated as a percentage of total leukocytes (Fig. 5A).

GS‐9688 induced MDSC activation, as determined by increased expression of CD80 (Fig. 5B) and altered the immunosuppressive potential of both subsets *in vitro*. Short‐term GS‐9688 exposure decreased expression of intracellular arginase‐I in PMN‐MDSCs, while simultaneously increasing its expression in M‐MDSCs, albeit to a much lower level than the PMN‐MDSCs (Fig. 5C). Expression of CD63, a measure of azurophilic granule mobilization to the cell surface, remained unchanged on PMN‐MDSCs but dramatically increased on M‐MDSCs (Fig. 5D). These data are suggestive of active degranulation of MDSC *in vitro* triggered by GS‐9688, supported by an increased release of extracellular arginase‐I into the culture media (Fig. 5E). In addition, we observed an increase in expression of the negative regulators galectin‐9 (on both subsets of MDSCs) and PD‐L1 (on the remaining M‐MDSCs), which could conceivably result in enhanced suppression of T‐cell immunoglobulin and mucin domain containing‐3 (Tim‐3)‐expressing or programmed cell death protein 1 (PD‐1)‐expressing HBV‐specific T cells^(^ ^6^, ^37^ ^)^ (Fig. 5F,G).

## Discussion

GS‐9688 is a potent TLR8 agonist in development for the treatment of CHB. A recent study in the woodchuck model of CHB revealed that a short, finite period of dosing with GS‐9688 is well tolerated and able to induce a sustained antiviral response.^(^ ^18^ ^)^ Here we evaluated the immunomodulatory effect of GS‐9688 on various immune cells *in vitro* using human PBMCs to understand its therapeutic potential for CHB.

Initial experiments confirmed GS‐9688 selectivity for TLR8, revealing the potent induction of immunomodulatory cytokines, such as IL‐12, by cDCs and MNPs, which was preserved in PBMCs from patients with CHB. In interrogating the effects on correlates of immune protection, we showed that GS‐9688 enhanced the frequency of HBV‐specific CD8^+^ T cells in a subset of patients. *In vitro* treatment with GS‐9688 boosted the production of antiviral cytokines, but not cytotoxicity, of HBV‐specific CD8^+^ T cells. The potential for GS‐9688 to enhance cellular mediators with antiviral potential in CHB was further evidenced by increased activation, improved cytolytic and noncytolytic effector function of NK cells, and MAITs. In the HBV‐infected liver, where NK cells are greatly enriched and where the virus‐specific T‐cell response is limited, it is possible that GS‐9688‐activated NK cells could perform a significant antiviral role through secretion of cytokines, and enhanced killing of infected hepatocytes. In line with other TLR8 agonists, we confirmed that the increase in antiviral activity by these effectors was driven by induction of IL‐12/IL‐18. IL‐12 has the potential to reverse mitochondrial defects of exhausted HBV‐specific CD8^+^ T cells, allowing for more efficient bioenergetics and enhanced antiviral functionality.^(^ ^38^, ^39^, ^40^ ^)^ Importantly, the therapeutic benefit of IL‐12 has been demonstrated in HBV transgenic mice, the woodchuck model of CHB, and in early clinical trials in patients with CHB.^(^ ^41^, ^42^, ^43^, ^44^ ^)^

Although promising, it is important to note that GS‐9688 did not induce an effective HBV‐specific CD8^+^ T‐cell response in PBMCs from all patients, which is typical of many immunotherapeutic strategies in development for CHB using diverse patient cohorts.^(^ ^6^, ^37^, ^45^ ^)^ This is reminiscent of the antiviral response to GS‐9688 in the woodchuck model of CHB, in which only half the animals treated with 3 mg/kg GS‐9688 had a sustained reduction in viral parameters.^(^ ^18^ ^)^ Consistent with the woodchuck study, the response of patient HBV‐specific CD8^+^ T cells *in vitro* was not associated with age, sex, extent of liver inflammation, or viral load. However, the magnitude of the *in vitro* response was associated with a low‐baseline HBsAg titer (<2,000 IU/mL). To elucidate the potential differences in responders and non‐responders, it is important to consider the contribution of potent immunoregulatory leukocytes implicated in suppressing T‐cell immunity. In this study we show increased expression of the death ligand TRAIL on NK cells, particularly on the CD56^bright^ subset driven by GS‐9688, which may further eliminate the already depleted pool of HBV‐specific T cells within the infected liver.^(^ ^8^ ^)^ Conversely, these TRAIL^+^NK cells may act as antifibrotic mediators in CHB, killing activated hepatic stellate cells in a TRAIL‐dependent manner or by producing hepatoprotective cytokines.^(^ ^46^ ^)^

A notable finding of this study was GS‐9688 modulation of immunosuppressive cells. GS‐9688 reduced the frequency of T_REG_, potent immunosuppressors implicated in CHB.^(^ ^31^ ^)^ Intriguingly, the decline in T_REG_ numbers was associated with a corresponding expansion and activation of cT_FH_. The observed GS‐9688‐driven increase in ICOS expression on cT_FH_ is significant, as ICOS is critical for IL‐21 production and the subsequent delivery of improved T‐cell help.^(^ ^47^, ^48^ ^)^ Although not studied here, we hypothesize that such an increase in cT_FH_ and T_FH_‐associated cytokines will improve immune control in CHB by modulating the frequency of memory B cells and the production of affinity‐matured class‐switched antibodies. Whether this decrease in T_REG_ and expansion and activation of cT_FH_ is sufficient to overcome the observed B‐cell dysfunction characteristic of patients with CHB will require further study.^(^ ^49^, ^50^ ^)^

Various other mechanisms have been identified that play a role in immune dysfunction in CHB, including the increased activity of MDSCs. Consistent with the literature,^(^ ^35^, ^36^ ^)^ we demonstrate the capacity of GS‐9688 to decrease the frequency of M‐MDSCs *in vitro*. However, this decrease was linked to an initial burst of arginase‐I release from PMN‐MDSCs, which may transiently limit the availability of the conditionally essential amino acid L‐arginine, acting as a crucial T‐cell rheostat.^(^ ^9^ ^)^ Of further significance is the capacity of GS‐9688 to induce expression of PD‐L1 and galectin‐9 on the remaining MDSCs, which could limit HBV‐specific CD8^+^ T cells expressing high levels of PD‐1 and Tim‐3.^(^ ^6^, ^37^ ^)^ Intrahepatic PD‐L1 and galectin‐9 levels are increased in viral hepatitis, and our data suggest that they may be further enhanced by TLR8‐induced IFN‐γ.^(^ ^37^, ^51^ ^)^ Collectively, these data raise the possibility that combination with checkpoint blockade may improve the antiviral response to GS‐9688 treatment.

GS‐9688 has good absorption and high first‐pass hepatic clearance, to limit systemic immune activation.^(^ ^17^ ^)^ After oral administration of GS‐9688, intestinal absorption is expected to induce TLR8 activation in the gut and liver, where the immune composition is markedly different.^(^ ^26^, ^52^, ^53^, ^54^ ^)^ It is likely that the secretion of immune mediators from the gut into the portal vein will in turn stimulate cells in the liver. Given the fact that the liver harbors a population of transcriptionally distant liver‐resident NK cells^(^ ^26^ ^)^ and memory CD8^+^ T cells^(^ ^53^, ^54^ ^)^ future studies will need to focus on characterizing the intrahepatic immune response to GS‐9688.

Although the therapeutic efficacy of GS‐9688 in combination with nucleos(t)ide treatment remains to be fully evaluated in patients with CHB, it was shown to be safe and generally well‐tolerated in a phase 2 placebo‐controlled study in virally suppressed patients, with a subset achieving HBsAg and/or HBeAg loss.^(^ ^55^ ^)^ While phase 2 studies are ongoing (ClinicalTrials.gov NCT03491553/NCT03615066), our work provides important insights into the immunomodulatory effects of GS‐9688 and has important implications for the rational design of combination studies with other immunomodulatory agents. Our data also raise the possibility that *in vitro* profiling of PBMC responses to GS‐9688 in patients with CHB may have utility for predicting therapeutic efficacy in patients.

## Author Contributions

L.J.P., S.D., S.P.F., and M.K.M. contributed to the study concept. O.E.A., E.J.C., L.J.P., L.P., C.F., S.D., S.P.F., and M.K.M. contributed to the experimental designs. E.J.C., J.D., S.K., D.R., D.P., R.C., H.J., H.M.S., S.L., A.P., C.F., and S.D. contributed to the data generation. O.E.A., E.J.C., L.J.P., L.P., C.F., H.J., and S.D. contributed to the data analysis. W.M.C.R. and M.K.M. obtained the clinical samples. O.E.A. and L.J.P. drafted the manuscript. All authors provided critical review of the manuscript.

## Supporting information

Supplementary Material
