# Supplementary material for: Therapeutic Potential of TLR8 Agonist GS‐9688 (Selgantolimod) in Chronic Hepatitis B: Remodeling of Antiviral and Regulatory Mediators
Source: Hepatology. 2021 Jun 20;74(1):55–71. doi: 10.1002/hep.31695 (PMC8436741; doi:10.1002/hep.31695)
Supplement: Supplementary file 1 — Supplementary Material [file HEP-74-55-s001.pdf]

Supplementary Material

Supplementary Figure Legends

Supplementary Figure 1

***In vitro* stimulation with GS-9688 induces comparable cytokine profiles healthy controls and CHB patients. a)** Summary data showing the mean ± SD for the minimal effective concentration (MEC; defined as the concentration of GS-9688 corresponding to a 3-fold induction above the DMSO, vehicle control), the EC_{50} (nM; defined as the concentration of GS-9688 giving 50% of a maximal cytokine response) and the E_{MAX} (pg/mL; the maximal concentration of cytokine produced) for each cytokine detected by Luminex in cell culture supernatants of PBMC isolated from healthy controls (HD; n=10) and CHB patients (n=10). **b)** Representative flow cytometric plots (left) and % $IFN\alpha^+$ (n=6) and **c)** CD40 mean fluorescence intensity (MFI; n=6) on circulating mononuclear phagocytes (MNP; $Lin1^+CD14^+$), conventional dendritic cells (cDC; $Lin1^-HLA\text{-}DR^+CD123^-CD11c^+$) and plasmacytoid dendritic cells (pDC; $Lin1^-HLA\text{-}DR^+CD123^+CD11c^-$) from GS-9688 treated healthy control PBMC (GS-9688 serial diluted from 10µM). Data represent the mean ± SEM.

Supplementary Figure 2

The *in vitro* effect of GS-9688 on HBV-specific $CD8^+$T-cells. HBV-specific $CD8^+$T-cells were expanded from PBMC by stimulation with pan-genotypic overlapping peptides spanning the HBV-core protein (OLP) in the presence of 0.1µM GS-9688 or vehicle control (DMSO). **a)** Sequential flow cytometric gating strategy used to identify $CD8^+$T-cells; defined as live, singlet lymphocytes, $CD3^+CD56^-CD4^-CD8^+$T-cells. FSC-A; forward scatter area, SSC-A; side scatter area. **b)** Representative flow cytometric plots (left) and % $CD107a^+$ $CD8^+$T-cells in response to HBV-core OLP in individual patients (n=19). Heat map denotes fold change of CD107a production upon treatment with GS-9688. **c)** Stratification of patient 'non-responders' and 'responders' (defined as a ≥1.2 fold increase in % $IFN\gamma^+$ HBV-specific T-cells in response to HBV core OLP in presence of 0.1µM GS-9688) by baseline serum alanine transaminase level (red; ALT IU/L; n=28) or HBV viral load (green; IU/mL; n=26).

Supplementary Figure 3

Effect of *in vitro* stimulation with GS-9688 on NK cell subsets. PBMC isolated from CHB patients were treated *in vitro* with GS-9688 (dose range) or vehicle control (DMSO) for 24hr. **a)** Sequential flow cytometric gating strategy used to identify NK cells; defined as live, singlet lymphocytes, $CD3^-CD56^+$ using CD16 to define $CD56^{bright}$ and $CD56^{dim}$ NK cells. FSC-A; forward scatter area, SSC-A; side scatter area. Representative flow cytometric plots and % CD69, HLA-DR and mean fluorescence intensity of CD38 expression (MFI) on **b)** $CD3^-CD56^{bright}$ NK cells (n=20) or **c)** $CD3^-CD56^{dim}$ NK cells (n=20). **d)** % $TNF\alpha^+$ (0.156µM; HC; n=8) and % $IFN\gamma^+$ (0.156µM; HC; n=8) NK cells stratified by $CD3^-CD56^{dim}$ and $CD3^-CD56^{bright}$. **e)** % $TRAIL^+$ $CD3^-CD56^{dim}$ and $CD3^-CD56^{bright}$ (n=20). Data represent the mean ± SEM. *p<0.5, **p<0.01; ***p<0.001; ****p<0.0001; Friedman test (ANOVA) with a Dunn's *post hoc* multiple comparisons test (**b-d**).

Supplementary Figure 4

***In vitro* stimulation with GS-9688 induces MAIT activation and effector function.** PBMC isolated from healthy controls were treated *in vitro* with GS-9688, using a single dose of 0.156µM or serial diluted from 10µM, or vehicle control (DMSO) for 18hr. **a)** Sequential flow cytometric gating strategy for the identification of mucosal-associated invariant T-cells (MAITs; live, singlet lymphocytes, $CD3^{+}TCR\gamma\delta^{-}CD161^{+}TCR\text{-}V\alpha7.2^{+}$). **b)** Representative flow cytometric plots (left) and % IFNγ^{+} (n=4), ± neutralisation of IL-12/IL-18 or isotype control (n=2) and **c)** % granzyme B^{+} (n=4) ± neutralisation of IL-12/IL-18 or isotype control (n=2). Circles and bars represent the mean ± SEM.

Supplementary Figure 5

***Impact of* GS-9688 on CD4+ T cell subsets *in vitro.* a)** Sequential flow cytometric gating strategy for the identification of $CD4^{+}$ regulatory T-cells (T_{REG}; live, singlet, $CD3^{+}CD8^{-}CD4^{+}CD25^{hi}CD127^{lo}FOXP3^{+}$) and circulating follicular helper $CD4^{+}$T-cells (cT_{FH}; live, singlet, $CD3^{+}CD56^{-}CD8^{-}CD4^{+}CXCR5^{+}PD\text{-}1^{+}$). FSC-A; forward scatter area, SSC-A; side scatter area. **b)** Summary data depicting % $CD4^{+}$ of total live lymphocytes (n=37).

Supplementary Figure 6

***In vitro* treatment with GS-9688 changes MDSC subset distribution in CHB patients. a)** Sequential flow cytometric gating strategy for MDSC subsets (PMN-MDSC; $CD11b^{+}CD33^{+}HLA\text{-}DR^{lo}CD14^{-}CD15^{+}$, M-MDSC; $CD11b^{+}CD33^{+}HLA\text{-}DR^{lo}CD14^{+}CD15^{-}$) in freshly isolated PBMC from CHB patients. **b)** Proportion of *ex vivo* MDSC subsets as a % of total immature myeloid cells ($CD11b^{hi}CD33^{+}$; n=26). **c)** PBMC from patients with CHB were stimulated with a single dose of 0.1µM GS-9688 or vehicle control (DMSO) for 18hr: % PMN-MDSC and M-MDSC as a % of total immature myeloid cells (n=26). Error bars represent the mean ± SEM. **p<0.01; ***p<0.001; ****p<0.0001. Wilcoxon Signed-rank *t* test (**b**-**c**).

Supplementary Table 1:

Clinical parameters for the cohort of CHB patients used to determine frequency and function of HBV-specific CD8^{+}T-cells *in vitro* upon GS-9688 treatment.

BLQ: below the level of quantification; ALT: alanine transaminase; N/A: not applicable

Donor	Gender (Male/ Female)	Age (years & Months)	HBeAg status	HBV DNA (IU/ml)	ALT (IU/L)	HBsAg titre (IU/ml)	Responder (Yes/No)	HLA Type
1	Male	25y 10mo	Negative	158489319	115	9626	Yes	N/A
2	Male	63y 2mo	Negative	BLQ	29	0	Yes	N/A
3	Male	36y 1mo	Negative	5370	28	834	Yes	N/A
4	Female	35y 4mo	Negative	70795	26	1147	Yes	N/A
5	Male	Unknown	Negative	BLQ	42	Unknown	Yes	N/A
6	Male	38y 9mo	Negative	1349	48	1233	Yes	N/A
7	Female	38y 6mo	Negative	955	30	1204	Yes	N/A
8	Male	77y 3mo	Negative	BLQ	29	596	Yes	N/A
9	Male	61y 3mo	Negative	661	37	1135	Yes	N/A
10	Female	59y 4mo	Negative	98	21	1581	Yes	N/A
11	Male	60y	Negative	1463	14	1418	Yes	N/A
12	Female	40y 4mo	Negative	490	16	Unknown	Yes	N/A
13	Male	67y	Negative	BLQ	27	400	Yes	N/A
14	Female	40y 3mo	Negative	794	20	Unknown	No	N/A
15	Male	41y 4mo	Negative	631	25	2400	No	N/A
16	Female	37y 2mo	Negative	105	22	Unknown	No	N/A
17	Male	38y 1mo	Negative	14454	66	161	No	N/A
18	Unknown	28y	Negative	3619	22	395	No	N/A
19	Male	29y	Positive	330542000	150	23945	No	N/A
20	Female	30y 9mo	Negative	BLQ	27	13652	No	N/A
21	Male	52y 9mo	Negative	4635	32	Unknown	No	N/A
22	Female	32y	Negative	BLQ	50	10100	No	N/A
23	Female	26y 1mo	Negative	630957344	37	100000	No	N/A
24	Male	30y 7mo	Negative	BLQ	35	3453	No	N/A
25	Female	40y 1mo	Negative	23	23	80	No	N/A
26	Male	19y 6mo	Negative	2337	79	53431	No	N/A
27	Male	43y 2mo	Negative	234423	109	820	No	N/A

28	Unknow n	36y	Negative	Unknown	27	14454	No	N/A
29	Male	56y 11mo	Negative	BLQ	Unknown	Unknown	N/A	A2
30	Male	59y 7mo	Negative	177828	17	4119	N/A	A2
31	Female	40y 11mo	Negative	5000	32	Unknown	N/A	A2
32	Female	Unknown	Negative	Unknown	Unknown	Unknown	N/A	
33	Male	Unknown	Negative	Unknown	Unknown	Unknown	N/A	A11/A24
34	Male	38y 5mo	Negative	BLQ	50	Unknown	NA	A2
35	Male	Unknown	Negative	9280	Unknown	Unknown	N/A	A2
36	Female	Unknown	Negative	39	Unknown	Unknown	N/A	A2/A24
37	Male	Unknown	Negative	6500	Unknown	Unknown	N/A	A2/A24
38	Female	Unknown	Negative	Unknown	Unknown	Unknown	N/A	A2/A11
39	Male	Unknown	Positive	Unknown	Unknown	Unknown	N/A	A11
40	Female	Unknown	Negative	39	Unknown	Unknown	N/A	
41	Female	38y 2mo	Positive	Unknown	76	Unknown	N/A	A2
42	Male	60y 1mo	Negative	3798	80	Unknown	N/A	A2
43	Male	Unknown	Negative	113	Unknown	Unknown	N/A	A2/A24
44	Female	40y 2mo	Negative	72	29	Unknown	N/A	A2
45	Male	32y 9mo	Negative	1500	38	Unknown	N/A	A2
46	Female	Unknown	Positive	Unknown	Unknown	Unknown	N/A	A2/A24
47	Female	58y 9mo	Negative	510000	46	Unknown	N/A	A2
48	Male	25y 10mo	Positive	322	27	Unknown	N/A	A2
49	Female	Unknown	Negative	Unknown	17	Unknown	N/A	A2
50	Male	23y 5mo	Negative	210	34	Unknown	N/A	A2
51	Male	41y 10mo	Negative	129609	47	Unknown	N/A	A2

Supplementary Table 2:

Details of HBV-specific HLA restricted ~~pentamers~~ multimers of the following specificities, used for identification of HBV-specific CD8$^+$T-cells

§ Supplied by the NIH Tetramer Core Facility, Emory University, Atlana, US

HLA haplotype	Multimer	Peptide sequence	Epitope	Fluorochrome
A*02:01	Pentamer	FLPSDFFPSV	HBcAg	PE
A*11:01	Pentamer	YVNVNMGLK	HBcAg	PE
A*24:02	Pentamer	EYLVSFGVW	HBcAg	PE
A*24:02	Pentamer	KYTSFPWLL	HBV polymerase	PE
A*02:01	Pentamer	FLLSLGIHL	HBV polymerase	PE
A*02:01	Pentamer	WLSLLVPFV	HBsAg	PE
A*02:01	Pentamer	GLSPTVWLSV	HBsAg	PE
A*02:01	Pentamer	FLLTRILTI	HBsAg	PE
A*02:01	Pentamer	FLPSDFFPSI	HBcAg	PE
A*02:01	Tetramer§	FLLTRILTI	HBsAg	APC
A*02:01	Tetramer§	WLSLLVPFV	HBsAg	APC
A*02:01	Tetramer§	LLVPFVQWFV	HBsAg	APC
A*02:01	Tetramer§	GLSPTVWLSV	HBsAg	APC
A*02:01	Tetramer§	KLHLYSHPI	HBV polymerase	APC
A*02:01	Tetramer§	GLSRYVARL	HBV polymerase	APC
A*02:01	Tetramer§	FLPSDFFPSV	HBcAg	APC

Supplementary Table 3:

Details of the reagents used in this study

**Antigen**	**Fluorochrome**	**Clone**	**Supplier**	**Dilution**
LIVE/DEAD™ Fixable Blue Dead Cell Stain	UV350	-	Thermo Fisher (L34961)	2:1000
LIVE/DEAD™ Fixable Aqua Dead Cell Stain	V405	-	Thermo Fisher (L34957)	1:400
**Myeloid Panel**				
Lin-1	FITC	UCHT1, HCD14, 3G8, HIB19, 2H7, HCD56	Biolegend (348801)	1:50
HLA-DR	V450	L243	BD Bioscience (642285)	1:50
CD11c	BV786	3.9	Biolegend (301601)	1:50
CD123	PerCP-Cy5.5	7G3	BD Bioscience (560904)	1:50
CD14	BV605	M5E2	BD Bioscience (564054)	1:50
IFNα	AF647	7N4-1	BD Bioscience (560088)	1:50
IL-12p40	PE	C11.5	Biolegend (501806)	1:50
TNFα	AF700	MAb11	BD Bioscience (557996)	1:50
CD40	APC-H7	5C3	BD Bioscience (561211)	1:50
CD86	PE-Cy7	2331	BD Bioscience (561128)	1:50
**$CD8^+$T-cell Panel**				
CD3	BUV395	UCHT1	BD Bioscience (564117)	1:100
CD4	APC-Cy7	RPA-T4	BD Bioscience (566319)	1:100
CD4	BV786	RPA-T4	BD Bioscience (740962)	1:100
CD8	AF700	RPA-T8	BD Bioscience (561453)	1:100
CD8	PerCP-Cy5.5	RPA-T8	BD Bioscience (560662)	1:100
CD56	PE-Cy7	NCAM16.2	BD Bioscience (335826)	1:100
HLA-DR	V500	G46-6	BD Bioscience (563083)	1:100
CD38	PerCP-Cy5.5	HIT2	Biolegend (303522)	1:100
TNFα	FITC	MAb11	BD Bioscience (554512)	1:100
IFNγ	BV421	B27	BD Bioscience (560371)	0.5:100
**NK Cell Panel**				
CD3	BV711	OKT3	Biolegend (317328)	1:100
CD3	BV605	OKT3	Biolegend (317321)	1:100

CD56	PE-Cy7	NCAM16.2	BD Bioscience (335826)	1:100
CD56	FITC	B159	BD Bioscience (562794)	1:100
CD69	BV786	FN50	Biolegend (310932)	1:100
HLA-DR	V500	G46-6	BD Bioscience (563083)	1:100
TRAIL (CD253)	BV421	RIK-2	BD Bioscience (56243)	2:100
CD38	PerCP-Cy5.5	HIT2	Biolegend (303522)	1:100
CD16	APC-Cy7	3g8	Biolegend (557758)	2:100
TNFa	FITC	MAB11	Biolegend (502905)	1:100
IFNγ	BV421	B27	BD Bioscience (560371)	0.5:100
IFNγ	APC	25723.11	BD Bioscience (655933)	1:100
Granzyme B	AF700	GB11	BD Bioscience (560213)	2:100
Perforin	BV510	DG9	Biolegend (308120)	1:100
CD107a	APC	H4A3	Biolegend (328620)	2.5µl/well
CD107a	PE-Cy7	H4A3	Biolegend (328617)	5µl/well
**MAITs Panel**				
CD3	PE	UCHT1	BD Biosciences (555333)	1:50
TCRγδ	FITC	11F2	BD Biosciences (347903)	1:50
CD161	BV605	HP-3G10	Biolegend (339916)	1:50
Vα7.2	AF647	3C10	Biolegend (351726)	1:50
IFNγ	APC-Cy7	B27	Biolegend (506524)	1:50
GranzymeB	AF700	GB11	BD Biosciences (560213)	1:50
**T_{REG} Panel**				
CD45	BUV805	H30	BD Bioscience (564915)	1:100
CD3	BV711	OKT3	Biolegend (317328)	1:100
CD4	APC-Cy7	RPA-T4	BD Bioscience (566319)	1:100
CD8	AF700	RPA-T8	BD Bioscience (561453)	1:100
CD25	PE-Cy7	Bc96	ThermoFisher (25-0259-42)	2:100
CD127	BV510	A019D5	Biolegend (351332)	1:100
FOXP3	BV421	206d	Biolegend (320123)	2:100
CD39	APC	A1	Biolegend (328210)	1:100
CTLA4	PE	BNI3	BD Bioscience (562742)	4:100
**Tfh Panel**				
CD56	PE-Dazzle	NCAM-HCD56	BD Bioscience (318348)	0.5:100
CD3	BUV805	UCHT1	BD Bioscience (612896)	1:100
CD4	BUV395	SK3	BD Bioscience (563550)	2:100

CXCR5	FITC	RF8B2	BD Bioscience (564624)	2:100
PD1	PE	EH12.2H7	Biolegend (329906)	1:100
ICOS	BV605	C398.4A	Biolegend (313537)	1:100
**MDSC Panel**				
CD45	BUV805	H30	BD Bioscience (564915)	1:100
CD56	PE-Dazzle	NCAM-HCD56	BD Bioscience (318348)	0.5:100
CD11b	PE-Cy7	Icrf44	Thermo Fisher (25-0018-42)	2:100
CD33	AF700	Wm-53	Thermo Fisher (56-0338-42)	3:100
HLA-DR	V450	G46-6	BD Bioscience (561359)	1:100
CD14	BV510	M5e2	Biolegend (301842)	2:100
CD3	BV711	OKT3	Biolegend (317328)	1:100
CD63	PerCP-Cy5.5	H5c6	Biolegend (561925)	2:100
CD15	APC	HI98	BD Bioscience (551376)	1:100
PD-L1	FITC	B7-H1	Biolegend (393605)	1:100
CD80	PE	2D10	Biolegend (305207)	1:100
Galectin-9	PE	9m1-3	Biolegend (348905)	1:100
Arginase-1	FITC	Met1-Lys322	R&D Systems (ic5868f)	5:100

Supp.Figure 1

a

Cytokine	MEC (nM)		EC_{50} (nM)		E_{max} (pg/ml)	
	HD	CHB	HD	CHB	HD	CHB
IL-12p40	29 ± 12	30 ± 14	217 ± 115	320 ± 151	9,108 ± 3,673	7,979 ± 3,977
IL-12p70	85 ± 61	98 ± 61	106 ± 122	310 ± 116	244 ± 157	200 ± 208
IL-18	65 ± 30	97 ± 70	219 ± 43	257 ± 87	153 ± 109	137 ± 119
IFNg	55 ± 14	92 ± 87	267 ± 85	322 ± 151	2,259 ± 2,558	1,067 ± 1,153
IFNa	168 ± 110	252 ± 135	999 ± 661	648 ± 394	38 ± 75	11 ± 15
TNFa	54 ± 12	51 ± 19	326 ± 137	319 ± 128	10,187 ± 6,594	10,673 ± 5,859
IL-1b	32 ± 13	47 ± 38	189 ± 72	198 ± 80	8,970 ± 2,618	9,614 ± 957
IL-6	18 ± 6	36 ± 51	151 ± 138	142 ± 109	26,413 ± 11,029	25,979 ± 10,978

b

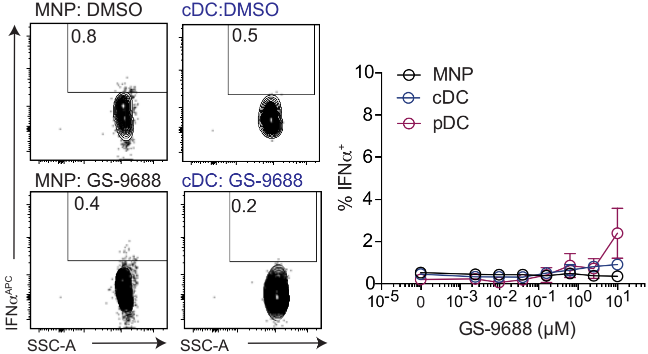

c

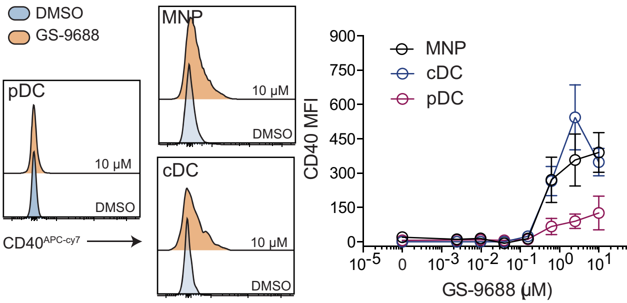

Supplementary Figure 2

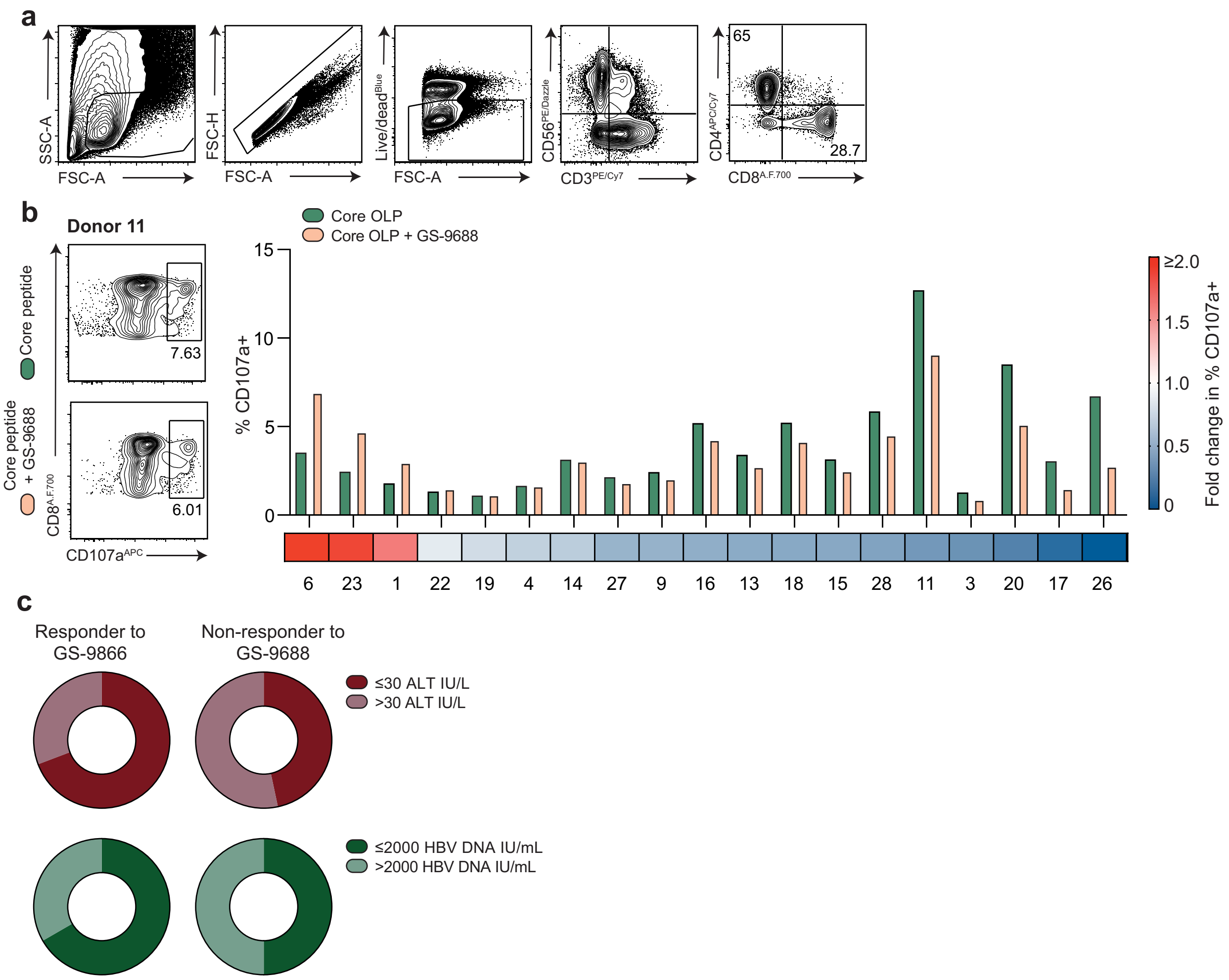

Supplementray Figure 3

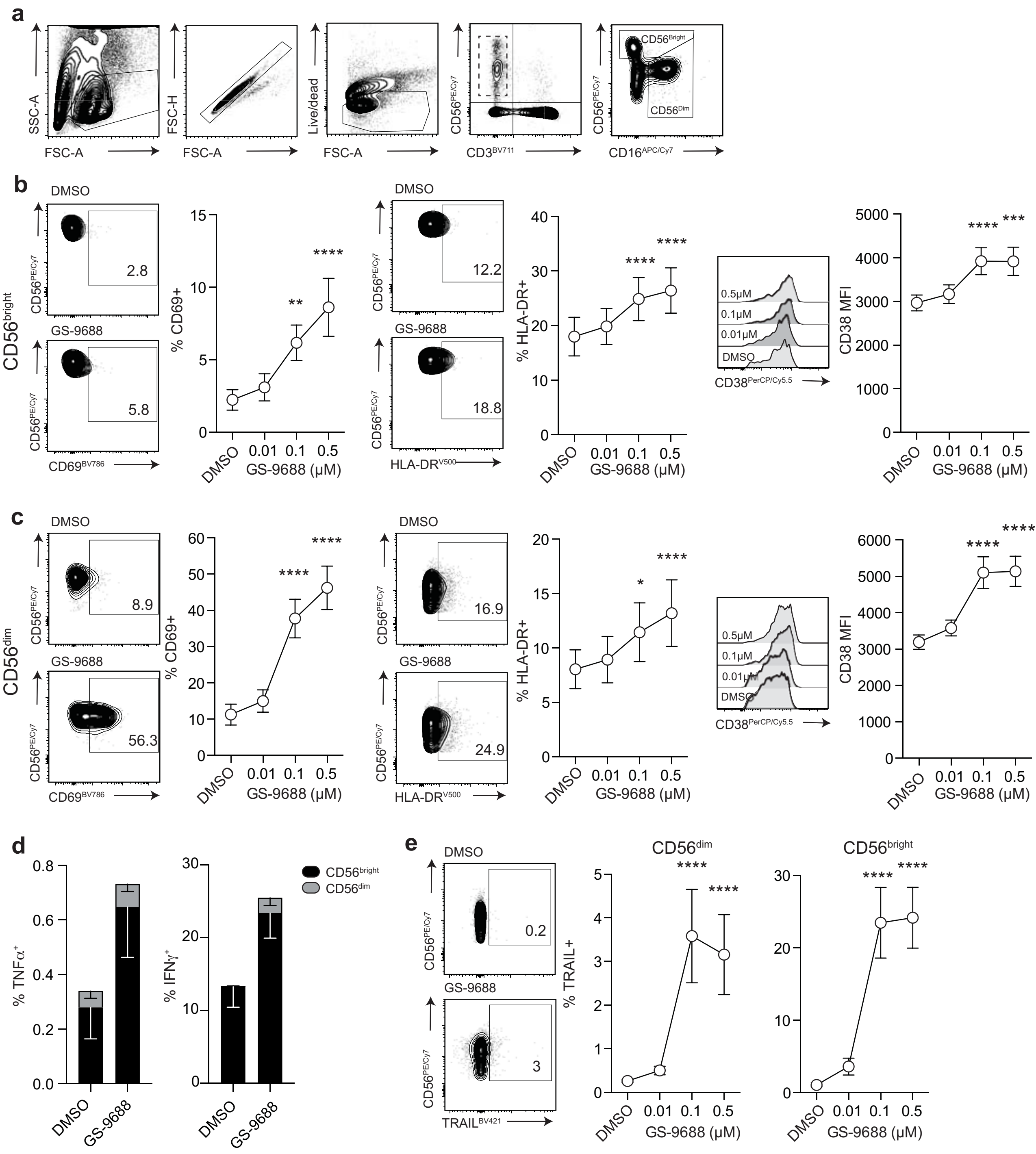

Supplementary Figure 4

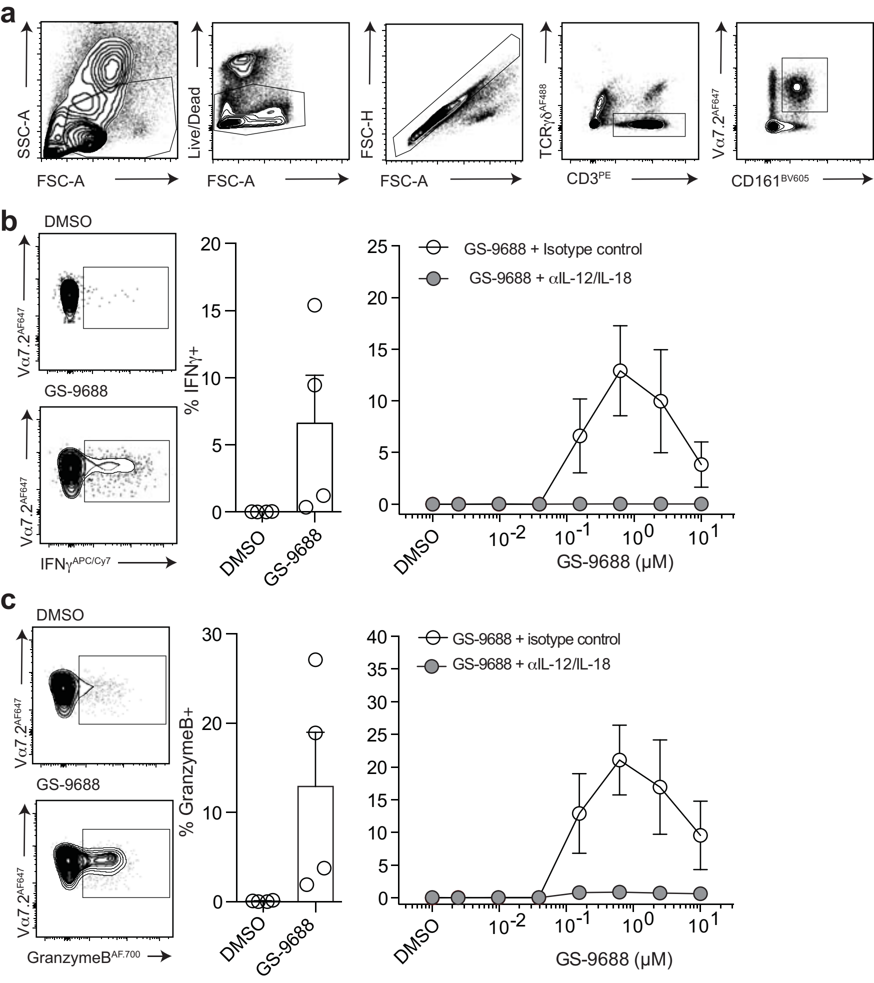

Supplementary Figure 5

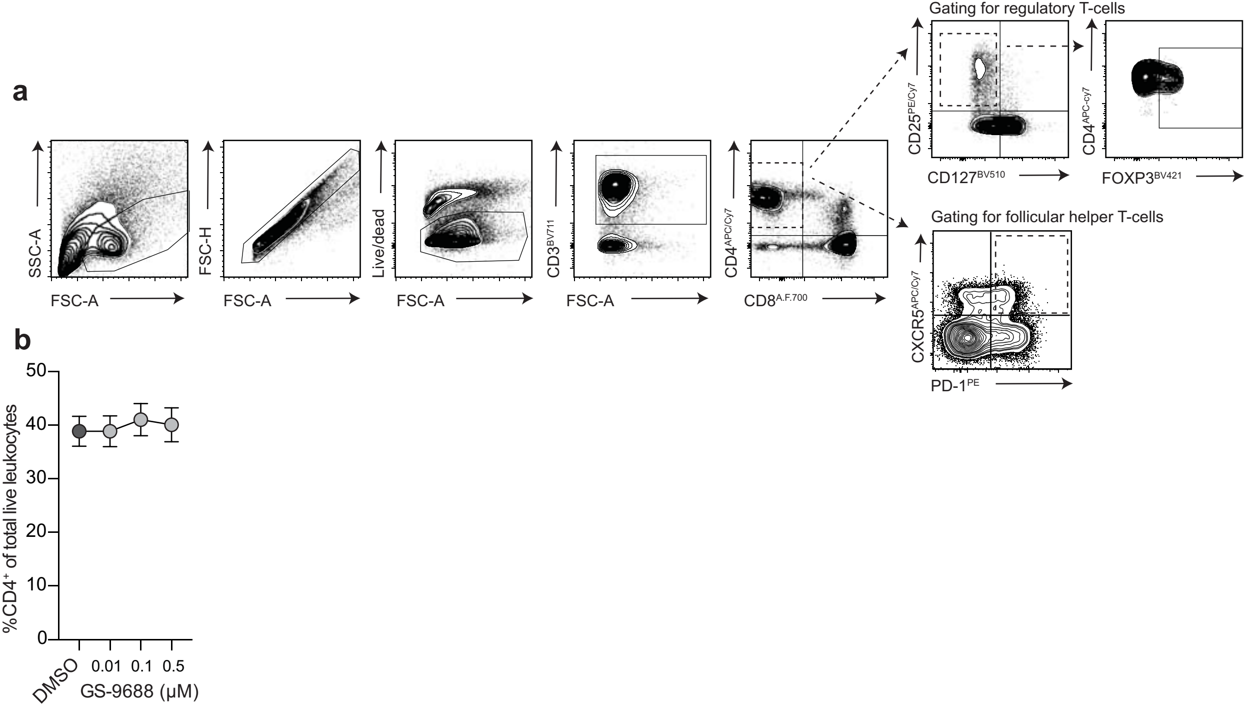

Supplementary Figure 6

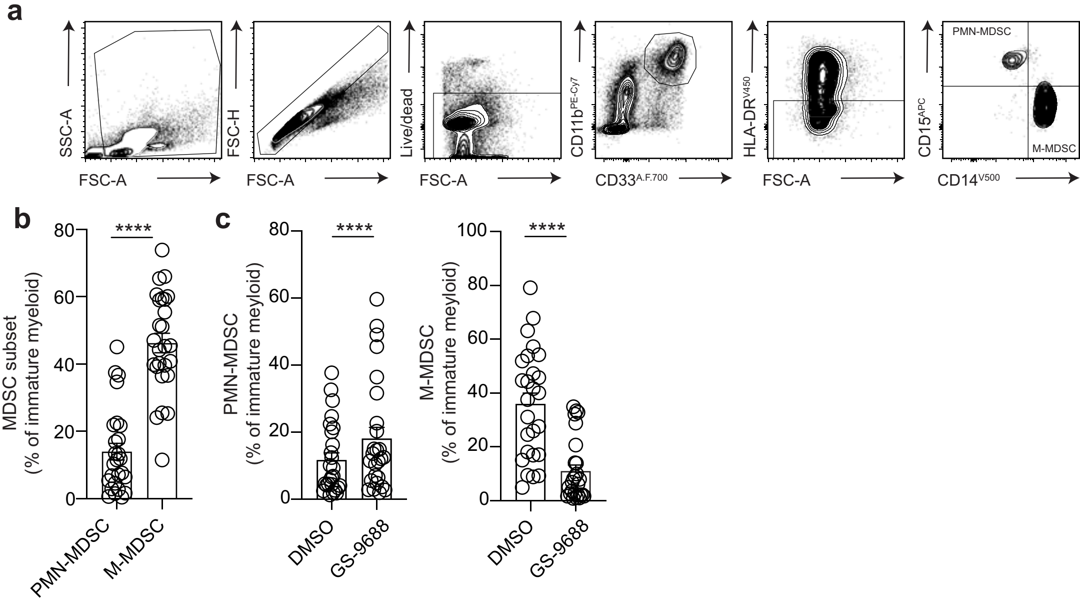

Supplementary Table 1:

Clinical parameters for the cohort of CHB patients used to determine frequency and function of HBV-specific $CD8^+$T-cells *in vitro* upon GS-9688 treatment.

BLQ: below the level of quantification; ALT: alanine transaminase; N/A: not applicable

Donor	Gender (Male/ Female)	Age (years & Months)	HBeAg status	HBV DNA (IU/ml)	ALT (IU/L)	HBsAg titre (IU/ml)	Responder (Yes/No)	HLA Type
1	Male	25y 10mo	Negative	158489319	115	9626	Yes	N/A
2	Male	63y 2mo	Negative	BLQ	29	0	Yes	N/A
3	Male	36y 1mo	Negative	5370	28	834	Yes	N/A
4	Female	35y 4mo	Negative	70795	26	1147	Yes	N/A
5	Male	Unknown	Negative	BLQ	42	Unknown	Yes	N/A
6	Male	38y 9mo	Negative	1349	48	1233	Yes	N/A
7	Female	38y 6mo	Negative	955	30	1204	Yes	N/A
8	Male	77y 3mo	Negative	BLQ	29	596	Yes	N/A
9	Male	61y 3mo	Negative	661	37	1135	Yes	N/A
10	Female	59y 4mo	Negative	98	21	1581	Yes	N/A
11	Male	60y	Negative	1463	14	1418	Yes	N/A
12	Female	40y 4mo	Negative	490	16	Unknown	Yes	N/A
13	Male	67y	Negative	BLQ	27	400	Yes	N/A
14	Female	40y 3mo	Negative	794	20	Unknown	No	N/A
15	Male	41y 4mo	Negative	631	25	2400	No	N/A
16	Female	37y 2mo	Negative	105	22	Unknown	No	N/A
17	Male	38y 1mo	Negative	14454	66	161	No	N/A
18	Unknown	28y	Negative	3619	22	395	No	N/A
19	Male	29y	Positive	330542000	150	23945	No	N/A
20	Female	30y 9mo	Negative	BLQ	27	13652	No	N/A
21	Male	52y 9mo	Negative	4635	32	Unknown	No	N/A
22	Female	32y	Negative	BLQ	50	10100	No	N/A
23	Female	26y 1mo	Negative	630957344	37	100000	No	N/A
24	Male	30y 7mo	Negative	BLQ	35	3453	No	N/A
25	Female	40y 1mo	Negative	23	23	80	No	N/A
26	Male	19y 6mo	Negative	2337	79	53431	No	N/A

27	Male	43y 2mo	Negative	234423	109	820	No	N/A
28	Unknown	36y	Negative	Unknown	27	14454	No	N/A
29	Male	56y 11mo	Negative	BLQ	Unknown	Unknown	N/A	A2
30	Male	59y 7mo	Negative	177828	17	4119	N/A	A2
31	Female	40y 11mo	Negative	5000	32	Unknown	N/A	A2
32	Female	Unknown	Negative	Unknown	Unknown	Unknown	N/A	
33	Male	Unknown	Negative	Unknown	Unknown	Unknown	N/A	A11/A24
34	Male	38y 5mo	Negative	BLQ	50	Unknown	NA	A2
35	Male	Unknown	Negative	9280	Unknown	Unknown	N/A	A2
36	Female	Unknown	Negative	39	Unknown	Unknown	N/A	A2/A24
37	Male	Unknown	Negative	6500	Unknown	Unknown	N/A	A2/A24
38	Female	Unknown	Negative	Unknown	Unknown	Unknown	N/A	A2/A11
39	Male	Unknown	Positive	Unknown	Unknown	Unknown	N/A	A11
40	Female	Unknown	Negative	39	Unknown	Unknown	N/A	
41	Female	38y 2mo	Positive	Unknown	76	Unknown	N/A	A2
42	Male	60y 1mo	Negative	3798	80	Unknown	N/A	A2
43	Male	Unknown	Negative	113	Unknown	Unknown	N/A	A2/A24
44	Female	40y 2mo	Negative	72	29	Unknown	N/A	A2
45	Male	32y 9mo	Negative	1500	38	Unknown	N/A	A2
46	Female	Unknown	Positive	Unknown	Unknown	Unknown	N/A	A2/A24
47	Female	58y 9mo	Negative	510000	46	Unknown	N/A	A2
48	Male	25y 10mo	Positive	322	27	Unknown	N/A	A2
49	Female	Unknown	Negative	Unknown	17	Unknown	N/A	A2
50	Male	23y 5mo	Negative	210	34	Unknown	N/A	A2
51	Male	41y 10mo	Negative	129609	47	Unknown	N/A	A2

Supplementary Table 2:

Details of HBV-specific HLA restricted ~~pentamers~~ multimers of the following specificities, used for identification of HBV-specific CD8^{+}T-cells

§ Supplied by the NIH Tetramer Core Facility, Emory University, Atlana, US

**HLA haplotype**	**Multimer**	**Peptide sequence**	**Epitope**	**Fluorochrome**
A*02:01	Pentamer	FLPSDFFPSV	HBcAg	PE
A*11:01	Pentamer	YVNVNMGLK	HBcAg	PE
A*24:02	Pentamer	EYLVSFGVW	HBcAg	PE
A*24:02	Pentamer	KYTSFPWLL	HBV polymerase	PE
A*02:01	Pentamer	FLLSLGIHL	HBV polymerase	PE
A*02:01	Pentamer	WLSLLVPFV	HBsAg	PE
A*02:01	Pentamer	GLSPTVWLSV	HBsAg	PE
A*02:01	Pentamer	FLLTRILTI	HBsAg	PE
A*02:01	Pentamer	FLPSDFFPSI	HBcAg	PE
A*02:01	Tetramer§	FLLTRILTI	HBsAg	APC
A*02:01	Tetramer§	WLSLLVPFV	HBsAg	APC
A*02:01	Tetramer§	LLVPFVQWFV	HBsAg	APC
A*02:01	Tetramer§	GLSPTVWLSV	HBsAg	APC
A*02:01	Tetramer§	KLHLYSHPI	HBV polymerase	APC
A*02:01	Tetramer§	GLSRYVARL	HBV polymerase	APC
A*02:01	Tetramer§	FLPSDFFPSV	HBcAg	APC

Supplementary Table 3:

Details of the reagents used in this study

Antigen	Fluorochrome	Clone	Supplier	Dilution
LIVE/DEAD™ Fixable Blue Dead Cell Stain	UV350	-	Thermo Fisher (L34961)	2:1000
LIVE/DEAD™ Fixable Aqua Dead Cell Stain	V405	-	Thermo Fisher (L34957)	1:400
**Myeloid Panel**				
Lin-1	FITC	UCHT1, HCD14, 3G8, HIB19, 2H7, HCD56	Biolegend (348801)	1:50
HLA-DR	V450	L243	BD Bioscience (642285)	1:50
CD11c	BV786	3.9	Biolegend (301601)	1:50
CD123	PerCP-Cy5.5	7G3	BD Bioscience (560904)	1:50
CD14	BV605	M5E2	BD Bioscience (564054)	1:50
IFNα	AF647	7N4-1	BD Bioscience (560088)	1:50
IL-12p40	PE	C11.5	Biolegend (501806)	1:50
TNFα	AF700	MAb11	BD Bioscience (557996)	1:50
CD40	APC-H7	5C3	BD Bioscience (561211)	1:50
CD86	PE-Cy7	2331	BD Bioscience (561128)	1:50
**$CD8^+$T-cell Panel**				
CD3	BUV395	UCHT1	BD Bioscience (564117)	1:100
CD4	APC-Cy7	RPA-T4	BD Bioscience (566319)	1:100
CD4	BV786	RPA-T4	BD Bioscience (740962)	1:100
CD8	AF700	RPA-T8	BD Bioscience (561453)	1:100
CD8	PerCP-Cy5.5	RPA-T8	BD Bioscience (560662)	1:100
CD56	PE-Cy7	NCAM16.2	BD Bioscience (335826)	1:100
HLA-DR	V500	G46-6	BD Bioscience (563083)	1:100
CD38	PerCP-Cy5.5	HIT2	Biolegend (303522)	1:100
TNFα	FITC	MAb11	BD Bioscience (554512)	1:100
IFNγ	BV421	B27	BD Bioscience (560371)	0.5:100
**NK Cell Panel**				
CD3	BV711	OKT3	Biolegend (317328)	1:100
CD3	BV605	OKT3	Biolegend (317321)	1:100

CD56	PE-Cy7	NCAM16.2	BD Bioscience (335826)	1:100
CD56	FITC	B159	BD Bioscience (562794)	1:100
CD69	BV786	FN50	Biolegend (310932)	1:100
HLA-DR	V500	G46-6	BD Bioscience (563083)	1:100
TRAIL (CD253)	BV421	RIK-2	BD Bioscience (56243)	2:100
CD38	PerCP-Cy5.5	HIT2	Biolegend (303522)	1:100
CD16	APC-Cy7	3g8	Biolegend (557758)	2:100
TNFa	FITC	MAB11	Biolegend (502905)	1:100
IFNγ	BV421	B27	BD Bioscience (560371)	0.5:100
IFNγ	APC	25723.11	BD Bioscience (655933)	1:100
Granzyme B	AF700	GB11	BD Bioscience (560213)	2:100
Perforin	BV510	DG9	Biolegend (308120)	1:100
CD107a	APC	H4A3	Biolegend (328620)	2.5µl/well
CD107a	PE-Cy7	H4A3	Biolegend (328617)	5µl/well
**MAITs Panel**				
CD3	PE	UCHT1	BD Biosciences (555333)	1:50
TCRγδ	FITC	11F2	BD Biosciences (347903)	1:50
CD161	BV605	HP-3G10	Biolegend (339916)	1:50
Vα7.2	AF647	3C10	Biolegend (351726)	1:50
IFNγ	APC-Cy7	B27	Biolegend (506524)	1:50
GranzymeB	AF700	GB11	BD Biosciences (560213)	1:50
**T_{REG} Panel**				
CD45	BUV805	H30	BD Bioscience (564915)	1:100
CD3	BV711	OKT3	Biolegend (317328)	1:100
CD4	APC-Cy7	RPA-T4	BD Bioscience (566319)	1:100
CD8	AF700	RPA-T8	BD Bioscience (561453)	1:100
CD25	PE-Cy7	Bc96	ThermoFisher (25-0259-42)	2:100
CD127	BV510	A019D5	Biolegend (351332)	1:100
FOXP3	BV421	206d	Biolegend (320123)	2:100
CD39	APC	A1	Biolegend (328210)	1:100
CTLA4	PE	BNI3	BD Bioscience (562742)	4:100
**Tfh Panel**				
CD56	PE-Dazzle	NCAM-HCD56	BD Bioscience (318348)	0.5:100
CD3	BUV805	UCHT1	BD Bioscience (612896)	1:100
CD4	BUV395	SK3	BD Bioscience (563550)	2:100

CXCR5	FITC	RF8B2	BD Bioscience (564624)	2:100
PD1	PE	EH12.2H7	Biolegend (329906)	1:100
ICOS	BV605	C398.4A	Biolegend (313537)	1:100
**MDSC Panel**				
CD45	BUV805	H30	BD Bioscience (564915)	1:100
CD56	PE-Dazzle	NCAM-HCD56	BD Bioscience (318348)	0.5:100
CD11b	PE-Cy7	Icrf44	Thermo Fisher (25-0018-42)	2:100
CD33	AF700	Wm-53	Thermo Fisher (56-0338-42)	3:100
HLA-DR	V450	G46-6	BD Bioscience (561359)	1:100
CD14	BV510	M5e2	Biolegend (301842)	2:100
CD3	BV711	OKT3	Biolegend (317328)	1:100
CD63	PerCP-Cy5.5	H5c6	Biolegend (561925)	2:100
CD15	APC	HI98	BD Bioscience (551376)	1:100
PD-L1	FITC	B7-H1	Biolegend (393605)	1:100
CD80	PE	2D10	Biolegend (305207)	1:100
Galectin-9	PE	9m1-3	Biolegend (348905)	1:100
Arginase-1	FITC	Met1-Lys322	R&D Systems (ic5868f)	5:100
